# Socioeconomic inequality, health inequity and well-being of transgender people during the COVID-19 pandemic in Nigeria

**DOI:** 10.1186/s12889-023-16482-1

**Published:** 2023-08-12

**Authors:** Morenike Oluwatoyin Folayan, Anna Yakusik, Amaka Enemo, Aaron Sunday, Amira Muhammad, Hasiya Yunusa Nyako, Rilwan Mohammed Abdullah, Henry Okiwu, Erik Lamontagne

**Affiliations:** 1 Department of Child Dental Health, Obafemi Awolowo University, Ile-Ife, Nigeria; 2 Nigeria Institute of Medical Research, Yaba, Lagos State Nigeria; 3 Joint United Nations Programme on HIV/AIDS, CH Geneva, Switzerland; 4 Nigeria Sex Workers Association, Kubwa, Nigeria; 5 African Network of Adolescent and Young Persons Development, Barnawa, Nigeria; 6 Northern Nigerian Transgender Initiative, Abuja, Nigeria; 7 Jami Al Hakeem Foundation Jimeta-Yola, Jimeta, Nigeria; 8 National Association of Persons with Physical Disability, Abuja, Nigeria; 9 YouthRise, Abuja, Nigeria; 10 School of Economics, Aix-Marseille University, Marseille, France

**Keywords:** LGBT, Transgender, Public health, Risk-taking, HIV, COVID-19, Vulnerability, Inequality, Socioeconomic, Well-being

## Abstract

**Background:**

We aimed to explore socioeconomic inequality, health inequity, and the well-being of transgender people during the COVID-19 crisis in Nigeria.

**Methods:**

Between June and December 2021, a cross-sectional survey was conducted collaboratively with community-based organisations in Nigeria. Participants living with or at risk of HIV were recruited voluntarily, online and face-to-face, using a combination of venue-based and snowball sampling. We assessed the association between gender identity (transgender and vulnerable cisgender women), and (i) socioeconomic inequality measured with socioeconomic status, social status, economic vulnerability, macrosocial vulnerability; (ii) health inequity measured with self-assessment of health, recency of HIV test, access to HIV and sexual and reproductive health services, gender-affirming care, financial and non-financial barriers to accessing health services; and (iii) well-being, measured with gender-based violence, mental health, psychoeconomic preferences. We used multivariable logistic regressions and controlled for interactions and confounders.

**Results:**

There were 4072 participants; 62% were under 30, and 47% reported living with HIV. One in ten (11.9%; *n* = 485) was transgender, and 56.5% reported living with HIV. Compared to vulnerable cisgender women, the results showed significantly higher odds (aOR:3.80) of disruption in accessing HIV services in transgender participants; gender-based violence (aOR:2.63); severe (aOR:2.28) symptoms of anxiety and depression. Among the barriers to accessing health and HIV services, transgender had three-time higher odds of reporting additional non-official fees compared to vulnerable cisgender women. The disclosure of their gender identity or sexual orientation was the most important non-financial barrier to accessing health services (aOR:3.16). Transgender participants faced higher housing insecurity (aOR: 1.35) and lower odds of using drugs (aOR:0.48). Importantly, they are more likely to have performed a recent HIV test and less likely to not know their HIV status (aOR:0.38) compared to vulnerable cisgender women.

**Conclusions:**

Socioeconomic inequality, health and well-being inequity in transgender people appear to be exacerbated by the COVID-19 pandemic in Nigeria. Interventions are necessary to mitigate socioeconomic challenges, address structural inequality, and ensure equitable access to health services to meet the Sustainable Development Goals for transgender people.

**Supplementary Information:**

The online version contains supplementary material available at 10.1186/s12889-023-16482-1.

## Introduction

 Transgender people is an umbrella term representing individuals whose gender identity differs from the sex they were assigned at birth. Despite substantial progress in the last decade, transgender people face social, legislative and health barriers. These challenges affect their physical and mental health as well as their well-being [1, 2]. In too many countries, punitive laws, practices, and policies against this population perpetuate stigma, discrimination, hate crimes, police abuse, torture, ill-treatment and family and community violence [3–5]. These may be structural (i.e., manifested in laws, policies, and institutionalised practices) or societal (i.e., due to rejection, mistreatment and social exclusion and lack of gender recognition by others). Both hinder the availability, access and uptake of HIV prevention, testing, treatment, care and support services and other sexual and reproductive health services, including gender-affirming care [6].

As defined by the World Health Organization (WHO), health inequities refer to systematic disparities in the health status experienced by various population groups [7]. These inequities have substantial social and economic consequences for both individuals and societies. The World Economic Forum provides a helpful distinction between equality and equity: Equality represents the ultimate objective, while equity denotes the means to achieve it. In other words, equality entails equal status, rights, and opportunities, whereas equity is about how we get there through fairness and impartiality [8, 9]. Following the above, in this study we referred to health inequity to describe an unfair, unjust, biased distribution of resource or services. One characteristics of health inequity is that it is avoidable. For example, no reason justifies that key and vulnerable women and girls or transgender people have poorer HIV and health services. Inequality on the other hand refers to inequality of opportunities among different population groups in choosing the type of life they want. Here, socioeconomic inequality denotes factors, rooted discriminatory practices and beliefs beyond personal control that could determine inequality of opportunity of transgender people compared to other vulnerable groups of adolescent girls and women. The socioeconomic inequality faced by key and vulnerable women and girls and transgender people stems from gender power relation and these hierarchical power relations stratify societies and perpetuate the social and economic inequalities, starting at childhood with lifelong consequences.

Transgender people experience more significant economic hardship, including lower employment rates and household incomes, higher poverty rates, and poor health outcomes [10]. Social and economic inequality is primarily driven by stigma, discrimination, and criminalisation [11]. Transgender people have limited access to education and employment, loss of opportunities for economic and social advancement, poor mental health outcomes, and increased vulnerability to HIV infection [12]. Stigma, discrimination, and criminalisation additionally lead to health inequity through barriers to accessing health care, including gender-affirming care [12] and increase the risk of violence, which can prompt or aggravate anxiety, depression, self-harm and suicidal behaviour among transgender people [13, 14].

The HIV epidemic disproportionately impacts transgender people. In 2021 UNAIDS estimated that the risk of acquiring HIV was 14 times higher among transgender people than among the rest of the adult population. In 2021, 12% of new HIV infections globally were among transgender women [15].

Stigma, discrimination, and criminalisation all compound to marginalising transgender and gender-diverse people, leading to the negation of the existence of gender-diverse persons. In 2021, twenty countries reported to UNAIDS that they formally criminalise or have prosecuted transgender people, and seventy countries reported that they outlaw homosexuality [15]. The situation faced by transgender people and other sexual and gender minorities in Nigeria is among the worst in the globe. The country was 153 out of 158 countries in term of homophobic climate index [16] and Pew Research Center found that only 7% of Nigeria’s population say homosexuality should be accepted, the lowest level of acceptance among the countries studied [17]. The situation is particularly worrying in the twelve Northern states of the country where death penalty legally apply for same-sex conduct [18].This leads to a severe lack of data on transgender and gender-diverse people and their health [19–21]. In many countries, national health information systems do not recognise transgender as a gender identity [22, 23], adding extra challenges for research in such contexts, including in Nigeria [24].

Acknowledging that the quality of the health care service delivery is generally poor in Nigeria [25], the inequity in health outcomes for transgender and gender-diverse people is further exacerbated by a unique set of health care needs such as sexual and reproductive health services, including gender-affirming care [26]. Stigma and discrimination based on gender identity often intersect with other forms of discrimination, for example, based on HIV status, drug use, involvement in sex work or socioeconomic status. These intersecting vulnerabilities are exacerbated by biological risks and social and sexual network-level risks for HIV and other sexually transmitted infections [27]. The stigma-related barriers include those created by health care providers who conflate gender identity and are not respectful of transgender people’s preferred pronouns; concern about being turned in to authorities; and outright refusal of access to health services [28].

Socioeconomic inequality and health inequity experienced by transgender and gender-diverse people in Nigeria and in Sub-Saharan Africa in general are insufficiently addressed. Some studies in Nigeria included transgender people as part of the lesbian, gay, bisexual, and transgender (LGBT) community [29, 30]. Though the LGBT community in Nigeria has been identified as a “hidden population” that experiences abuse, rejection, and marginalisation from healthcare providers [31, 32], the specific needs of transgender people differ from that of other sexual minority groups. The high prevalence of HIV among transgender people in Nigeria [33] and stigma-related barriers to accessing HIV services [34] highlight the need for better integrating transgender-specific services in the country.

The COVID-19 pandemic reportedly creates new and exacerbates pre-existing inequality, including in Nigeria [35–39]. In this study, we aimed to assess the effects of the COVID-19 pandemic on transgender socioeconomic inequality and health inequity during Nigeria’s second and third wave of the pandemic. Our research design is underpinned by the intersecting forms of health inequity, social and economic inequality and the marginalisation of transgender people. We adopted the Intersectionality Research for Transgender Health Justice research framework and posited that socioeconomic inequality and health inequity affecting transgender populations are produced by structures, institutional systems, and socio-structural processes that affect the design of programmes, development of policies, and the institution of actions for transgender health justice [40]. Considering the context described above, we chose to compare the situation faced by transgender people with other key and vulnerable women and girls in terms of HIV infection. These population groups are defined in the 2021 United Nations Political Declaration on HIV and AIDS and ending inequality [41].

The study objectives were (i) to identify the differences in the sociodemographic profile of transgender people in Nigeria compared with that of cisgender women at high risk of acquiring HIV or living with HIV; (ii) to assess how the COVID-19 pandemic affected access to HIV and sexual and reproductive health services, including gender-affirmative care; (iii) to identify the differential effect of the COVID-19 pandemic on the engagement in sex work by cisgender women and transgender people; and (iv) to assess the well-being of transgender people during the COVID-19 pandemic.

We assumed that (i) the sociodemographic profile of cisgender women and transgender people sex workers would not differ significantly in Nigeria; (ii) the COVID-19 pandemic severely affects access to HIV and sexual and reproductive health services, including gender-affirmative care; (iii) the well-being of transgender people is worse than that of cisgender women during the COVID-19 pandemic.

## Methods

### Ethical consideration

The study protocol was approved by the Institute of Public Health Research Ethics Committees from Obafemi Awolowo University (IPH/OAU/12/1692) and the ethics committee in Lagos State (LS/C.350/S.1/215), Anambra State (MH/AWK/M/321/363), Adamawa State (ADHEC07/06/2021), Akwa Ibom State (MH/PRS/99/Vol.V/994), Benue State (MOH/STA/208/VOL.1/183) and Kaduna State (MOD/ADM/774/VOL.1/1008). A waiver for parental consent for adolescents 15–17 years old was obtained for this non-intrusive sexual and reproductive health research by the Institute of Public Health, Obafemi Awolowo University Health Research Committee, in accordance with the national guidelines on sexual and reproductive health research conducted with adolescents [42].

### Study design, study sites and study population

In 2021, UNAIDS joined community-based organisations and research partners to undertake cross-sectional surveys of girls and women living with or at risk of HIV in Nigeria to gauge the COVID-19 pandemic’s impact on their lives. Participants from Adamawa, Akwa-Ibom, Anambra, Benue, Kaduna, and the Lagos States, Enugu, Gombe, Nasarawa and Niger States were recruited from June to December 2021 during the second and third waves of COVID-19 in Nigeria using a combination of two non-probabilistic, purposive sampling methods, a venue-based and snowball methods. For the venue-based sampling, community leads from civil society organisations, community-based organisations and networks at the state level discussed the study with community members. Study participants were recruited in the CBOs and networks premises and received a weblink to the survey to fill the questionnaire independently using phone, tablet, or computer. If the participant had issues accessing the internet, an internet device was provided by the interviewer. The latter were also trained to offer computer-assisted personal interviewing (CAPI) if participants had literacy issues. For snowball sampling, respondents who completed the survey were given coupons to invite up to five peers who may not have been connected to community organisations. Finally, an online river sampling method was implemented through the community leaders who reached out to their members in Enugu, Gombe and Niger States. The community organisations posted the survey link on social media (Facebook, Twitter, Instagram and WhatsApp groups) or email networks and invite peers to take the survey. The data were collected using a web-based survey platform, LimeSurvey™. Data were stored encrypted on the European server, compliant with EU Regulation 2016/679 on the General Data Protection Regulation (GDPR). The survey was available in English. Keywords in the questionnaires were translated into Yoruba, Igbo, Hausa, and specific dialects or local languages predominant in the States studied. Translation into local dialects were made in consultation with community leaders participating in the project.

The target populations were cisgender adolescent girls and women and transgender people living with or at high risk of acquiring HIV, including women who sell sex, women who engage in transactional sex, women living with disability, migrants, refugees and displaced persons, and women who use psychoactive substances.

Participants had to provide their informed consent and their age before entering to the survey’s modules. Underage participants and those not providing their informed consent were automatically excluded from the survey tool. Details of the study methodology had been reported in prior studies [43, 44].

### Sample size

The pre-survey minimum sample size for this study was set at 60 valid respondents per each target study population group in each of the target states. It corresponds to a minimum sample size of 2160 participants. From the statistical modelling perspective, we tried to have a minimum of 108 valid participants per vulnerability category at the national level, enabling us to perform regressions with up to eight predictors with a minimum probability level (p-value) of 0.05.

### Study instrument

The study questionnaire contained validated instruments for collecting survey data among women and key populations. The questionnaire was first reviewed for content validity, pretested and harmonised with standard indicators and protocol checklists used in behavioural surveillance. The data was collected using LimeSurvey™, a web-based survey platform. The survey was administered in English, but keywords in the questionnaires were translated into Yoruba, Igbo or Hausa and to specific dialects or local languages that were predominant in the target states. This approach was used when implementing national surveys because of the diversity of languages in Nigeria [45]. Participants had to provide their written informed consent before filling out the online questionnaire by ticking a checkbox.

### Study procedures

National networks of the different target populations were involved in designing and implementing the study protocol. Community entry leads for adolescent girls and women living with HIV were identified by the African Network of Adolescent and Young Person’s Development (ANAYD). The National Association of Persons with Physical Disability (NAPWPD) identified the community entry leads for females living with a disability. The National Sex Workers Association (NSWA) identified the community entry leads for sex workers. YouthRise identified the community entry leads for female drug users. The Northern Nigerian Transgender Initiative identified the community entry leads for transgender women, and Jami Al Hakeem Foundation identified the community entry leads for migrants and refugees.

The leadership of the Networks reviewed and suggested revisions to the study protocol, made the decisions on the states for the data collection, conducted community entry programs and supported the participants’ recruitment process using a combination of venue-based and snowball convenience sampling methods. Additional participants were recruited through the river sampling method. Recruitment strategies were discussed and adjusted to ensure the diversity of recruitment locations (rural, urban, and semi-urban) and socioeconomic strata. Trained field workers were linked to community entry leads which helped with the targeted recruitment of 108 valid participants per target group, per State.

The field workers consented to the study participants and provided them with a web link to the survey. The personal electronic device used by respondents for the survey could only be used once, thereby limiting multiple survey responses by a respondent. The questionnaires were filled independently by participants using the phone, tablet, or computer-assisted self-interviewing. When participants had literacy challenges, the interviewer offered computer-assisted personal interviewing. Interested study participants who came to the study venue and had no electronic device could access the survey tool using the field worker’s electronic device.

Physical distancing and other COVID-19 prevention measures were ensured at the data collection venues. Respondents were provided with a face mask and hand sanitiser during the data collection process. Respondents who participated in the study were given airtime vouchers for data/internet usage valued at $1.70 (N1000) and coupons to invite up to five peers not connected to the local community organisations. Additional participants were recruited through the river sampling method.

### Independent variable

Participants were asked to define their sex at birth (female, male, intersex) and how they would primarily define their gender identity. Possible answers were “Woman”, “Transgender woman (male to female)”, “Transgender man (female to male) “, “Other”, “I don’t know”, “I cannot or do not wish to answer this question”. Transgender participants were those who identified their gender as transgender woman or man and had opposite sex at birth or were born intersex. Cisgender women are those participants whose sex at birth is female or intersex and identify themselves as women. We created a dichotomic outcome variable with cisgender women (0) and transgender people (1).

### Study dependent variables

We present below the independent variables for each of the three models developed in the study: socioeconomic inequality, health inequity, and well-being.

#### Socioeconomic inequality

*Subjective socioeconomic status was assessed using the McArthur scale *[46] with the following question: “Think of a ladder representing where people stand in your country. At the top of the ladder are the people who are the best off. At the bottom are the people who are the worst off. Where would you place yourself on this ladder at this moment?“ Possible answers were a 10-item Likert scale ranging from “1: among those having the least money, least education and least respected jobs or no job”, to “10 among those having the most money, most education and most respected jobs”. The participants’ responses were grouped per tercile. See supplementary files for the description of the SES terciles.

*Subjective social status was assessed using the McArthur scale *[46] with the following question: “Now, think of a ladder representing where people stand in your local community. Where would you place yourself on this ladder at this moment?“ Possible answers were a 10-item Likert scale ranging from “1: lowest standing in my community”, to “10: highest standing in my community”. The participants’ responses were grouped per tercile. See supplementary files for the description of the SSS terciles.

##### Economic vulnerability

This was measured using three parameters: food, financial and housing insecurity. We performed several preliminary tests and the three selected variables on financial vulnerability appeared to be both valid and robust proxy for economic vulnerability. Food insecurity was measured using a self-reported response to the question: “Since the COVID-19 crisis began, do you eat less or skip meals because there was not enough money for food?“. The financial vulnerability was measured using self-reported responses to the question “Do you have enough money today to cover the daily expenses of today and tomorrow?“ Housing insecurity was measured using self-reported answers to the question “Since the COVID-19 crisis began, did you move in with other people, even for a little while, because of financial problems?“ The possible response options to the three questions were “yes”, “no”, “I cannot or do not wish to answer the question”. The “yes” responses were classified as facing either food, financial or housing insecurity during COVID-19. The questions were adapted from the questionnaire by Santos and colleagues [47].

##### Macrosocial vulnerability

The survey also collected specific information on using psychoactive drugs, engaging in sex work and engaging in transactional sex. These categories are not mutually exclusive. Participants can identify themselves with more than one category. We adjusted the model to account for interactions between each of these categories.

#### Health inequity

Health inequity was studied through three groups of variables reflecting self-measures of general health and HIV, the impact of the COVID-19 crisis on access to health services and the barriers to accessing health services during COVID-19.

##### Self-assessment of health

Participants were asked the following question to self-assess their health: “In general, how would you rate your health?“. Possible answers were a 5-Likert scale from excellent to poor. This is standard self-rated health used in national and global surveys [48].

##### HIV status

HIV status was self-reported in response to the question, “Do you know your HIV status?“ The response options were “I am HIV-positive”, “I am HIV-negative”, “I do not know my HIV status”, and “I cannot or do not want to answer this question”. Considering that people do not test for the same reason they do not disclose their HIV status [49–51], the “I don’t know” and “I do not want to answer this question” were combined into a single response.

##### Access to HIV and sexual and reproductive health services

Respondents were asked if the COVID-19 pandemic impacted their attendance at the health facilities for HIV prevention, treatment and care-related services, referred to as HIV services [52]. Participants were also asked if they had access to abortion, family planning, sexually transmitted infection treatment and gender-based violence services, otherwise referred to as sexual and reproductive health services [53]. Respondents were asked if the COVID-19 pandemic impacted their attendance at the health facilities for any HIV and sexual and reproductive health services when needed. Respondents had the option of ticking “yes”, “no”, or “not needed”. An indication of the inability to access any of these services indicated the respondent’s failure to access an HIV or sexual and reproductive health service. The responses were dichotomised into “yes” and “no/not needed”. A “yes” response indicated poor access to either of the services [53].

##### Financial and non-financial barriers to HIV and sexual and reproductive health services

Study participants were asked if costs, expenditures or other (non-financial) reasons prevented them from accessing HIV or sexual and reproductive health services needed during the COVID-19 pandemic. For this study, data were extracted for two financial and three non-financial variables significantly associated with the risk of poor access to HIV and sexual and reproductive health services during the COVID-19 pandemic in Nigeria [22]. These were: (1) paying fees at the clinic or hospital; (2) the additional unofficial fees to the cost of care; (3) “worried people could disclose one’s sexual orientation”; (4) “was humiliated at my last visit for HIV service”; and (5) “faced improper treatment [violence, insult, or discrimination] the last time I went for HIV service”.

##### Access to transgender-specific services

Respondents were asked if the COVID-19 pandemic impacted their attendance at the transgender-specific services; limited their ability to access medications and hormones specific to trans healthcare; ability to access non-medical supplies such as make-up, shaving supplies, wigs, breast forms; access to therapy or counselling services for transgender-specific support including peer support groups; and access gender-affirmation or transition-related surgery. Respondents had the option of ticking “yes”, “no”, or “not needed”. The responses were dichotomised into “yes” and “no/not needed”. A “yes” response indicated poor access to either of the services. The questions were constructed by transgender researchers, implemented in comparable surveys [54], and validated by Nigerian transgender community organisations.

#### Well-being

##### Survivor of gender-based violence

 The participants were asked about their experience of gender-based violence during the time of the COVID-19 crisis. The possible answers to the question “Do you feel that you are currently experiencing”: were: “More violence than before the COVID-19 crisis”; “The same level of violence as before the COVID-19 crisis”; “Less violence than before the COVID-19 crisis”; “I am not experiencing any violence”; “I cannot or do not wish to answer this question”. Participants who answered they were facing more, same, or less violence were grouped and categorised as survivors of gender-based violence. Those who reported not experiencing any violence was categorised as not victims of gender-based violence.

##### Mental health

Psychological distress was measured using the Patient Health Questionnaire-4, a 4-point Likert-type scale. The validated instrument screens for depression and anxiety [55]. It has been used to screen psychological distress during the COVID-19 pandemic in Nigeria [56]. The possible scores ranged from 0 to 12. Psychological distress was categorised into none (0–2), mild (3-5), moderate (6-8) and severe (9-12). For this study, the Cronbach alpha score was 0.888.

##### Psycho-economic characteristics

 We considered three psycho-economic factors of participants: their willingness to take risks, their time preference and their trust in other people. We used the following validated standardised questions from the behavioural economics literature [57–59]. The willingness to take risks was measured with the question How willing are you to take risks, in general? Possible answers were an 11-item Likert scale from 0, very unwilling, to 10, very willing. Time preference was measured with the question “How willing are you to give up something that is beneficial for you today in order to benefit more from that in the future?“ Possible answers were an 11-item Likert scale from 0, very unwilling to do so, to 10, very willing to do so. The trust in other people was measured with the following assessment: “How well does the following statement describe you as a person: I assume that people have only the best intentions”. Possible answers were according to an 11-item Likert scale from “0, does not describe me at all” to “10, describes me perfectly”. The three variables were categorised in terciles.

### Confounders

For each model, we controlled with sociodemographic variables such as age (grouped as adolescent girls and young women (15–24-year-old), adult (25–44-year-old) and older adult (≥ 45 years), educational achievement (none or primary level, secondary level, and post-secondary level), and a dichotomised geospatial location variable (urban, rural).

### Covariates

Participants were asked about their history of engagement in sex work (yes/no). For those who had engaged in sex work, they were asked if there was a change in sex work engagement due to the pandemic (no change/there was a change); what type of change (engaged more in sex work, engaged in sex work online, earned less money per customer, take more risks or do things beyond my comfort, engage in more condomless sex, stopped sex work, engaged in less sex work). Respondents had the option of ticking a response (“yes” or “no”) for each of the questions asked.

### Data analysis

We first performed a descriptive analysis of all the study variables for each of the three dimensions that impact transgender people’s living conditions: socioeconomic inequality, health inequity, and well-being. Next, we tested the association between the dependent and the independent and confounding variables using Pearson’s chi-square test. In a second step, we built three multivariable logistic regression models to determine the association between being a transgender person and the determinants of each of the three dimensions described above. We controlled for confounders and enabled interactions between variables that could be linked, such as being a survivor of gender-based violence and mental health. We performed post-estimations tests, including likelihood ratio chi-square, and we controlled for the hypothesis of a null value for the independent variables for each model. In addition, we performed additional analyses of variance, margins, collinearity, goodness-of-fit. Finally, we controlled for specification errors, and tested whether or not the interactions between potentially related variables (for example between transactional sex and sex work) were relevant in improving the model. Statistical significance was taken at a p-value equal to or less than 0.05. Statistical analyses were performed using Stata 16.

## Results

In this section we present the sociodemographic characteristics of the sample, followed by descriptive statistics of the each of the three dimensions that impact transgender people’s living conditions: socioeconomic inequality, health inequity, and well-being. The results of the logistic regressions are then presented, per dimension.

### Sociodemographic characteristics

A total of 4072 participants living with or at risk of HIV completed the survey in Nigeria. The sociodemographic profile of study participants is presented in Table 1. The majority (86%, *n* = 3587) of respondents were key and vulnerable cisgender women and 485 (11.9%) transgender people. Most (54.0%) cisgender women participants were adults (25–44-year-old) though most of the transgender participants (53.8%) were adolescents and young people (15–24-year-old). The majority of cisgender (44.5%) and transgender (49.7%) participants had completed secondary school and lived in a town (49.7% of cisgender women and 62.3% of transgender people).

Table 1 shows that the majority of participants (42.7%) declared themselves in the middle tercile regarding socioeconomic status but in the lower tercile (46%) regarding social standing in their community.

Since the COVID-19 pandemic started, most participants (74.0%) faced food insecurity and were financially vulnerable (80.5%). The phenomenon was more prevalent among vulnerable cisgender women than transgender participants. More transgender people (26.0%) faced housing insecurity than vulnerable cisgender women (18.6%). In terms of macrosocial categories of vulnerability, 23.8% of vulnerable women reported using drugs, a higher share than transgender participants (17.2%). A larger share of transgender participants (65.3%) engaged in sex work, that is 25% points more compared to vulnerable cisgender women. Similarly, a larger share (72.5%) of transgender reported engaging in transactional sex, that is 26.2% points more than among vulnerable cisgender women.

**Table 1:** Socioeconomic characteristics of transgender people and women at risk of or living with HIV

Variables	Total		Cisgender Women		Transgender	
	(*N* = 4 072)		(*N* = 3587)		(*N* = 485)	
	**%**	**n**	**%**	**n**	**%**	**n**
Individual characteristics						
Age groups	Pearson chi2(2) = 81.8676 Pr = 0.000					
Adolescent girls & young women (15–24)	38,4%	1565	36.4%	1304	53.8%	261
Adults (25–44)	53,0%	2160	54.0%	1938	45.8%	222
Older adults (45+)	8,5%	347	9.6%	345	0.4%	2
Highest level of education	Pearson chi2(2) = 186.7638 Pr = 0.000					
From none to primary education	32,9%	1341	36.1%	1296	9.3%	45
Secondary education	45,2%	1839	44.5%	1598	49.7%	241
Post-secondary or University degree	21,5%	874	18.9%	679	40.2%	195
Missing	0,4%	18	0.4%	14	0.8%	4
Location	Pearson chi2(3) = 124.3402 Pr = 0.000					
A large city	9,8%	401	8.6%	308	19.2%	93
A town	51,2%	2084	49.7%	1782	62.3%	302
A village	35,3%	1438	37.8%	1357	16.7%	81
A farm or isolated house	2,9%	118	3.2%	114	0.8%	4
Missing	0,8%	31	0.7%	26	1.0%	5
Socioeconomic inequality						
Subjective socioeconomic status	Pearson chi2(2) = 35.7852 Pr = 0.000					
Lower tercile	35,6%	1450	36.5%	1308	29.3%	142
Middle tercile	42,7%	1737	43.0%	1544	39.8%	193
Higher tercile	19,4%	790	18.0%	647	29.5%	143
Missing	2,3%	95	2.5%	88	1.4%	7
Subjective social standing status	Pearson chi2(2) = 57.1549 Pr = 0.000					
Lower tercile	46,0%	1875	48.0%	1720	32.0%	155
Middle tercile	29,2%	1189	28.7%	1031	32.6%	158
Higher tercile	22,6%	921	21.1%	756	34.0%	165
Missing	2,1%	87	2.2%	80	1.4%	7
Food insecurity	Pearson chi2(1) = 52.8119 Pr = 0.000					
No	22,1%	901	20.4%	732	34.8%	169
Yes	74,0%	3012	75.7%	2716	61.0%	296
Missing	3,9%	159	3.9%	139	4.1%	20
Financial vulnerability	Pearson chi2(1) = 15.6085 Pr = 0.000					
No	16,3%	662	15.4%	552	22.7%	110
Yes	80,5%	3278	81.2%	2913	75.3%	365
Missing	3,2%	132	3.4%	122	2.1%	10
Housing insecurity	Pearson chi2(1) = 14.4568 Pr = 0.000					
No	77,0%	3137	77.8%	2792	71.1%	345
Yes	19,4%	792	18.6%	666	26.0%	126
Missing	3,5%	143	3.6%	129	2.9%	14
Macrosocial categories of vulnerability						
People who use drugs (*n* = 3674, missing = 398, chi2(1) = 9.07 *p* = 0.003)	23,0%	845	23.8%	771	17.2%	74
Engaged in sex work (*n* = 3676, missing = 396, chi2(1) = 99.67 *p* = 0.000)	43,0%	1579	40.0%	1298	65.3%	281
Engaged in transactional sex (*n* = 3675, missing = 397, chi2(1) = 103.53 *p* = 0.000)	49,4%	1814	46.3%	1505	72.5%	309

The survey enabled the identification of several risk factors for those engaging in sex work at the time of COVID-19. Table 2 provides information on the differences between cisgender women and transgender people on how the pandemic impacted their sex work. For most risk factors, the difference between women and transgender engaging in sex work was not significant. It is worth noting that transgender people were earning less money per customer and engaged more in online sex during the COVID-19 pandemic.

**Table 2:** Risk factors for those engaging in sex work

Variables	Total		Women		Transgender	
	(*N* = 4072)		(*N* = 3587)		(*N* = 485)	
	%	(n)	%	(n)	%	(n)
Engaged in sex work	Pearson chi2(1) = 99.67 Pr = 0.000					
No	51.5%	2097	54.3%	1948	30.7%	149
Yes	38.8%	1579	36.2%	1298	57.9%	281
Missing	9.7%	396	9.5%	341	11.3%	55
Has the COVID-19 crisis influenced your engagement in sex work?						
Was there a change in your sex work?	Pearson chi2(1) = 0.1267 Pr = 0.722					
No change in my sex work engagement	40.5%	1650	38.2%	1370	57.7%	280
There was a change in my sex work engagement	2.8%	115	2.6%	94	4.3%	21
Not applicable (no sex work)	56.7%	2307	59.2%	2123	37.9%	184
I engage in more sex work	Pearson chi2(1) = 0.0738 Pr = 0.786					
No	25.1%	1021	23.7%	849	35.5%	172
Yes	18.3%	744	17.1%	615	26.6%	129
Not applicable (no sex work)	56.7%	2307	59.2%	2123	37.9%	184
I engage in sex work online	Pearson chi2(1) = 25.2202 Pr = 0.000					
No	37.4%	1521	35.9%	1289	47.8%	232
Yes	6.0%	244	4.9%	175	14.2%	69
Missing and not applicable (no sex work	56.7%	2307	59.2%	2123	37.9%	184
I earn less money per customer	Pearson chi2(1) = 34.7251 Pr = 0.000					
No	30.3%	1233	27.3%	980	52.2%	253
Yes	13.1%	532	13.5%	484	9.9%	48
Not applicable (no sex work)	56.7%	2307	59.2%	2123	37.9%	184
I take more risks or do things beyond my comfort	Pearson chi2(1) = 3.4575 Pr = 0.063					
No	35.7%	1453	33.3%	1194	53.4%	259
Yes	7.7%	312	7.5%	270	8.7%	42
Not applicable (no sex work)	56.7%	2307	59.2%	2123	37.9%	184
I engage in more condomless sex	Pearson chi2(1) = 0.0115 Pr = 0.915					
No	40.8%	1663	38.4%	1379	58.6%	284
Yes	2.5%	102	2.4%	85	3.5%	17
Not applicable (no sex work)	56.7%	2307	59.2%	2123	37.9%	184
I have stopped sex work	Pearson chi2(1) = 0.2925 Pr = 0.589					
No	41.8%	1703	39.3%	1411	60.2%	292
Yes	1.5%	62	1.5%	53	1.9%	9
Not applicable (no sex work)	56.7%	2307	59.2%	2123	37.9%	184
I engage in less sex work	Pearson chi2(1) = 7.6909 Pr = 0.006					
No	35.9%	1463	33.4%	1197	54.8%	266
Yes	7.4%	302	7.4%	267	7.2%	35
Not applicable (no sex work)	56.7%	2307	59.2%	2123	37.9%	184

Table 3 provides the characteristic of the participants in terms of health inequity. Overall, 1475 (41.1%) vulnerable cisgender women considered themselves in good health, while 164 (33.8%) transgender people considered themselves in very good health. Also, while 1652 (46.1%) cisgender women self-reported living with HIV, the self-reported HIV prevalence was significantly higher among the transgender participants (56.5%; *n* = 274). A larger share (84.5%) of transgender participants reported a recent (within the last 12 months) HIV test compared to vulnerable women (68.3%).

**Table 3:** Health inequity characteristics of transgender people and women at risk of or living with HIV

Variables		Total		Women			Transgender	
		(*N* = 4072)		(*N* = 3587)			(*N* = 485)	
		**%**	**n**	**%**	**n**		**%**	**n**
Health Inequity								
Self-assessment of health status		Pearson chi2(4) = 83.1818 Pr = 0.000						
Poor		4,0%	161	4.0%	143	3.7%		18
Fair		16,4%	669	16.9%	607	12.8%		62
Good		39,6%	1612	41.1%	1475	28.2%		137
Very good		27,7%	1127	26.8%	963	33.8%		164
Excellent		10,6%	432	9.2%	331	20.8%		101
Missing		1,7%	71	1.9%	68	0.6%		3
HIV status		Pearson chi2(2) = 40.2114 Pr = 0.000						
HIV-negative		39,6%	1611	39.8%	1429	37.5%		182
HIV-positive		47,3%	1926	46.1%	1652	56.5%		274
I don’t know or do not wish to answer		11,3%	460	12.4%	443	3.5%		17
Missing		1,8%	75	1.8%	63	2.5%		12
Recency of last HIV test		Pearson chi2(2) = 67.2409 Pr = 0.000						
Never		9,1%	370	10,1%	362	1,6%		8
More than 12 months		18,8%	766	19,8%	712	11,1%		54
Less or equal to 12 months		70.2%	2860	68.3%	2450	84.5%		410
Missing		1,9%	76	1,8%	63	2,7%		13
Did COVID-19 situation affect your access to sexual and reproductive health (SRH) services when it was needed		Pearson chi2(1) = 79.5929 Pr = 0.000						
No / Not needed		65,0%	2646	67.0%	2404	49.9%		242
Yes		29,0%	1180	26.5%	951	47.2%		229
Missing		6,0%	246	6.5%	232	2.9%		14
Did COVID-19 situation affect your access to HIV services when it was needed		Pearson chi2(4) = 185.2278 Pr = 0.000						
No / Not needed		52,9%	2154	56.0%	2009	29.9%		145
Yes		41,7%	1698	38.3%	1374	66.8%		324
Missing		5,4%	220	5.7%	204	3.3%		16
Barriers to accessing SRH or HIV services								
Fees at the clinic or hospital		Pearson chi2(1) = 4.5588 Pr = 0.033						
No		73,9%	3011	73.4%	2633	77.9%		378
Yes		26,1%	1061	26.6%	954	22.1%		107
The additional non-official fees		Pearson chi2(1) = 28.5533 Pr = 0.000						
No		94,6%	3853	95.3%	3419	89.5%		434
Yes		5,4%	219	4.7%	168	10.5%		51
I’m worried people could discover my gender identity or sexual orientation		Pearson chi2(1) = 262.6809 Pr = 0.000						
No		87,9%	3579	90.9%	3262	65.4%		317
Yes		12,1%	493	9.1%	325	34.6%		168
Last time I was humiliated		Pearson chi2(1) = 2.1513 Pr = 0.142						
No		97,1%	3955	97.3%	3489	96.1%		466
Yes		2,9%	117	2.7%	98	3.9%		19

Concerning access to health services during the COVID-19 pandemic, 229 (47.2%) of transgender people faced a disruption in their access to sexual and reproductive health services compared to 951 (26.5%) of cisgender women participants. Access to HIV prevention, treatment and care services was disrupted for 324 (66.8%) transgender people, which was 18% points higher when compared to cisgender women (38.3%, *n* = 1374). More transgender people than cisgender women experienced disruption of health services.

On the one hand, significantly more vulnerable women than transgender people identified fees paid at the clinic or hospital as a financial barrier to accessing health and HIV services (*p* = 0.000). On the other hand, significantly more transgender people than women identified non-official fees as a financial barrier to accessing health and HIV services (*p* = 0.001). The only non-financial barrier to accessing health and HIV services was the fear of disclosing one’s sexual orientation or gender identity. Significantly more transgender people than cisgender women identified this as a barrier (*p* = 0.000).

Table 4 shows that 188 (31.9%) of transgender people reported disrupted access to medication and hormones, 161 (27.3%) had disrupted access to therapy or counselling services for transgender-specific support; 140 (23.7%) reported a disruption in accessing non-medical supplies; and 91 (15.4%) had a disruption in access to planned gender-affirmation or transition-related surgery mainly because of cancelled or non-rescheduled (5.9%; *n* = 35), rescheduled and completed (4.9%, *n* = 29), or future rescheduled (4.4%, *n* = 26) cancellations.

**Table 4:** Access to transgender-specific services during the COVID-19 in Nigeria

Services		Among transgender people	
		**(N)**	**%**
In the past 3 months, has the COVID-19 situation limited your ability to access medications and hormones specific to your trans healthcare?			
No		182	30.8%
Yes		188	31.9%
Do not use or not applicable		189	32.0%
Do not know or do not want to answer		11	1.9%
Missing		20	3.4%
In the past 3 months, has the COVID-19 situation limited your ability to access to non-medical supplies? (example: make-up, shaving supplies, wigs, breast forms, etc.)			
No		255	43.2%
Yes		140	23.7%
Do not use or not applicable		158	26.8%
Do not know or do not want to answer		15	2.5%
Missing		22	3.7%
In the past 3 months, has the COVID-19 situation limited your ability to access to therapy or counselling services for transgender-specific support? (including peer support groups)			
No		223	37.8%
Yes		161	27.3%
Do not use or not applicable		169	28.6%
Do not know or do not want to answer		15	2.5%
Missing		22	3.7%
In the past 3 months, has the COVID-19 situation limited your ability to access gender-affirmation or transition-related surgery?			
No		248	42.0%
Yes		91	15.4%
Do not use or not applicable		212	35.9%
Do not know or do not want to answer		20	3.4%
Missing		19	3.2%
If yes, how was your surgery or surgeries impacted?			
Cancelled without rescheduling and/or delayed indefinitely		35	5.9%
Rescheduled and completed		29	4.9%
Rescheduled for the future with the same provider		14	2.4%
Rescheduled for the future with a different provider		12	2.0%
Not applicable		480	81.4%
Missing		20	3.4%

Table 5 shows that transgender people with severe symptoms of anxiety and depression (22.3%) were 8% points higher than vulnerable cisgender women (13.9%). Also, though the majority of cisgender women at high risk did not face gender-based violence (67.2%), the percentage of transgender people who did not face gender-based violence is 24% points lower (43.3%). 19% of transgender people have faced more violence since the COVID-19 pandemic than cisgender women at high risk (10%). In addition, transgender people had a higher mean value with a smaller standard error than vulnerable women for the statistically significant psycho-economic markers - willingness to take risks, time preference, and trust.

**Table 5:** Well-being characteristics of transgender people and women at risk or living with HIV in Nigeria

Variables	Total		Women		Transgender	
	(*N* = 4 072)		(*N* = 3587)		(*N* = 485)	
	**%**	**n**	**%**	**n**	**%**	**n**
Well-being						
Syndromes of anxiety or depression	Pearson chi2(3) = 32.2983 Pr = 0.000					
None	23,2%	943	24.0%	861	16.9%	82
Mild symptoms of anxiety and depression	28,2%	1149	28.6%	1025	25.6%	124
Moderate symptoms of anxiety and depression	23,9%	972	23.4%	841	27.0%	131
Severe symptoms of anxiety and depression	14,9%	607	13.9%	499	22.3%	108
Missing	9,8%	401	10.1%	361	8.2%	40
Experience of gender-based violence	Pearson chi2(3) = 110.4198 Pr = 0.000					
I am not experiencing any violence	64,3%	2620	67.2%	2410	43.3%	210
Less violence than before the COVID-19 crisis	7,1%	291	6.6%	235	11.5%	56
The same level of violence as before the COVID-19 crisis	9,7%	397	8.9%	318	16.3%	79
More violence than before the COVID-19 crisis	11,0%	449	10.0%	357	19.0%	92
Missing	7,7%	315	7.4%	267	9.9%	48
Happiness (life satisfaction)	Pearson chi2(2) = 38.2091 Pr = 0.000					
Suffering	33,8%	1378	24.0%	861	16.9%	82
Struggling	29,6%	1207	28.6%	1025	25.6%	124
Thriving	33,5%	1364	23.4%	841	27.0%	131
Missing	3,0%	123	13.9%	499	22.3%	108
Psycho-economic preferences						
Willingness to take risks	Pearson chi2(10) = 97.0318 Pr = 0.000					
N (n missing)	3971	101	3498	(89)	473	(12)
Mean (standard deviation)	5,52	3.10	5.35	(3.11)	6.80	(2.69)
Time preference	Pearson chi2(10) = 51.1998 Pr = 0.000					
N (n missing)	3935	(137)	3464	(123)	471	(14)
Mean (standard deviation)	6,23	2,74	6.14	(2.76)	6.88	(2.54)
Trust in other people	Pearson chi2(10) = 66.0549 Pr = 0.000					
N (n missing)	3974	(98)	3502	(85)	472	(13)
Mean (standard deviation)	5,47	2,81	5.40	(2.82)	6.05	(2.64)

### Socioeconomic inequality

Table 6 shows that transgender people had a significantly higher adjusted odds ratio (aOR 1.55, 95%CI 1.25–1.92) of being young (15-24-year-old), 1.4 times higher odds of having completed a post-secondary or university degree (aOR 1.42, 95%CI 1.12–1.80), and almost two times higher odds (aOR 1.926, 95%CI 1.48–2.51) of living in urban areas when compared to vulnerable women. In addition, transgender people had significantly higher odds (aOR 1.44, 95%CI 1.08–1.93) of being in the lower socioeconomic tercile (SES) compared to those in the middle tercile; higher odds (aOR 1.35, 95%CI 1.05–1.72) of facing housing insecurity; and lower odds (aOR 0.72, 95%CI 0.56–0.92) of having faced food insecurity compared to cisgender women. There was no statistically significant difference in the risk for financial vulnerability between the two populations. In terms of macrosocial categories of vulnerability, transgender people have significantly lower odds of using psychotropic drugs (aOR 0.48, 95%CI 0.24–0.98) but had more than 2.6 times higher odds of engaging in sex work (aOR 2.89, 95%CI 1.52–5.48) and transactional sex (aOR 2.74, 95%CI 1.85–4.07) compared to cisgender women.

**Table 6:** Socioeconomic inequality faced by transgender people during the COVID-19 pandemic in Nigeria

Variables	aOR	*p*-value	95% CI	
Age groups				
Adolescent girls and young women (15–24)	1.545	0.000	1.246	1.915
Adults (25–44)	(base)			
Older adults (45+)	0.609	0.117	0.328	1.133
Highest level of education				
From none to primary education	0.300	0.000	0.215	0.420
Secondary education	(base)			
Post-secondary or University degree	1.417	0.004	1.118	1.795
Location				
Living in urban areas	1.926	0.000	1.480	2.505
Subjective socioeconomic status (SES)				
Lower tercile	1.443	0.013	1.080	1.928
Middle tercile	(base)			
Higher tercile	1.001	0.993	0.727	1.380
Subjective social standing status (SSS)				
Lower tercile	0.813	0.172	0.605	1.094
Middle tercile	(base)			
Higher tercile	0.932	0.669	0.677	1.285
Economic vulnerability				
Food insecurity	0.716	0.008	0.560	0.916
Financial vulnerability	0.991	0.950	0.744	1.320
Housing insecurity	1.347	0.017	1.054	1.723
Macrosocial categories of vulnerability				
Using drugs	0.482	0.043	0.237	0.978
Engaged in sex work	2.888	0.001	1.524	5.475
Engaged in transactional sex	2.742	0.000	1.847	4.069
Constant	0.083	0.000	0.053	0.129
N	3343			
Log-likelihood	-1168.13			
Likelihood ratio chi2(19)	312.520			
prob > chi2	0.000			

### Health inequity

Table 7 shows that transgender participants had significantly higher odds of reporting being in good (aOR 1.25, 95%CI 1.12–1.39) health. In terms of recency of their last HIV test, transgender people have a reduction of 40% of their odds (95%CI 0.37–0.99) of having an HIV test older than 12 months and a reduction of 62% reduction of their odds (95%CI 0.19–0.78) of never having performed an HIV test compared to having a recent (within the last 12 months) HIV test. We controlled the latter variable on HIV testing for interactions with two variables measuring the disruption of health services. There was no significant difference in the odds of disrupted access to sexual and reproductive health (aOR 0.73, 95%CI 0.58–0.92) for transgender and vulnerable cisgender women participants (aOR 1.33, 95% CI 0.83–02.13). However, transgender people had more than three-time higher odds (aOR 3.80, 95%CI 2.86–5.05) of reporting disruption in accessing HIV services - either prevention, care or treatment - due to the COVID-19 pandemic.

**Table 7:** Health inequity faced by transgender people during the COVID-19 pandemic in Nigeria

Variables	aOR	*p*-value	95% CI	
Age groups				
Adolescent girls & young women (15–24)	1.515	0.000	1.232	1.864
Adults (25–44)	(base)			
Older adults (45+)	0.573	0.068	0.315	1.041
Highest level of education				
From none to primary education	0.322	0.000	0.236	0.438
Secondary education	(base)			
Post-secondary or University degree	1.319	0.016	1.053	1.653
Location				
Living in urban areas	1.943	0.000	1.506	2.506
Self-assessment of health status	1.250	0.000	1.123	1.391
Recency of last HIV test				
Never	0.381	0.008	0.187	0.775
More than 12 months	0.603	0.045	0.367	0.989
Less or equal to 12 months	(base)			
Did COVID-19 crisis have an impact on accessing health services when it was needed				
Access to HIV services when it was needed	3.801	0.000	2.859	5.052
Access to SRH services when it was needed	1.328	0.239	0.828	2.130
Barriers to accessing SRH or HIV services				
Fees at the clinic or hospital	0.767	0.088	0.566	1.040
The additional non-official fees	3.009	0.001	1.568	5.775
I’m worried people could discover my gender identity or sexual orientation	3.164	0.000	2.327	4.301
Last time I was humiliated	1.374	0.514	0.530	3.566
Constant	0.049	0.000	0.035	0.069
N	3963			
Log likelihood	-1299.67			
Likelihood ratio chi2(30)	614.69			
prob > chi2	0.000			

Among the barriers to accessing health and HIV services, there was no significant difference in the odds of identifying healthcare centres’ standard fees as a barrier between the two groups. (aOR 0.77, 95% CI 0.57–1.04). On the other hand, it appears that transgender had three-time higher odds (95%CI 1.57–5.78) of reporting additional non-official fees as a financial barrier compared to cisgender women. Finally, transgender participants had more than three-time higher odds of identifying disclosure of their gender identity or sexual orientation (aOR 3.16, 95%CI 2.33–4.30) as a barrier to access to health services compared to cisgender women. We controlled for eventual interactions between the different barriers.

### Mental well-being

Table 8 shows that transgender people had 2.63 higher odds of being survivors of gender-based violence than cisgender women (95%CI 1.59–4.33). Also, they had higher odds of moderate (aOR 1.61, 95%CI 1.09–2.37) to severe (aOR 2.28, 95%CI 1.50–3.47) symptoms of anxiety and depression compared to vulnerable cisgender women. Regarding psycho-economic markers, transgender people reported slightly higher odds of willingness to take risks (aOR 1.12, 95%CI 1.07–1.17) and trust in other people (aOR 1.06, 95%CI 1.02–1.10). They also had a lower time preference (aOR 0.95, 95%CI 0.90–0.99) than vulnerable women.

**Table 8:** Well-being reported by transgender people and women at risk of or living with HIV

Variables	aOR	*p*-value	95% CI	
Age groups				
Adolescent girls & young women (15–24)	1.716	0.000	1.398	2.105
Adults (25–44)	(base)			
Older adults (45+)	0.598	0.090	0.330	1.083
Highest level of education				
From none to primary education	0.320	0.000	0.233	0.440
Secondary education	(base)			
Post-secondary or University degree	1.264	0.041	1.009	1.582
Location				
Living in urban areas	2.439	0.000	1.892	3.145
Survivor of gender-based violence	2.626	0.000	1.593	4.328
Syndromes of anxiety or depression				
None	(base)			
Mild symptoms of anxiety and depression	1.332	0.132	0.917	1.934
Moderate symptoms of anxiety and depression	1.611	0.016	1.094	2.372
Severe symptoms of anxiety and depression	2.283	0.000	1.500	3.474
Psychoeconomic decision-making preferences				
Willingness to take risks	1.118	0.000	1.071	1.167
Time preference	0.946	0.025	0.901	0.993
Trust in other people	1.060	0.005	1.018	1.103
Constant	0.022	0.000	0.013	0.036
N	3606			
Log-likelihood	-1262.56			
Likelihood ratio chi2(15)	385.150			
prob > chi2	0.000			

Regarding statistical methods and tests, we examined *i*) the patterns of missing variables, *ii*) the independence of the two population groups, and *iii*) post-estimation tests.

i) Except for the informed consent and the age question, participants could skip any question they do not feel comfortable with or cannot answer. The number and percentage of missing participants are presented in each table above. The average share of missing participants is 3.45%. The share of participants skipping questions was the highest for questions related to symptoms of anxiety and depression (9.8%) and gender-based violence (GBV) (9.7%). Although there is no published standard on the missing rates for these questions, we found that these percentages are comparable to other surveys conducted among key and vulnerable population groups [60, 61]. We performed, for each of the three models (socioeconomic inequality, health inequity, and mental well-being), Little’s chi-squared tests for missing completely at random (MCAR) and covariate-dependent missingness (CDM) on the complete sample and on the transgender people sample. It appeared that the skipping patterns were comparable between the two samples for variables with a relatively higher number of missing responses, such as *engaging in sex work*, *engaging in transactional sex* and *using drugs* in the second model and variables *mental health* and *gender-based violence* in the third model. The Little’s chi-square tests were conclusive for all the above groups. In other words, we rejected the assumption that variables were not missing completely at random (CMAR), and found no covariate-dependent missingness (CDM). Finally, the regressions were performed using the criteria of total completion, i.e., we considered only those participants who informed all variables in each model, and we did not impute missing variables.
ii) We tested the independence of the two population groups for each independent variable and the whole model. As presented in Table 1, the Pearson chi2 test of independence showed that the differences observed between key and vulnerable cisgender women and transgender people were statistically significant for each independent variable. The sample size exceeds the minimum required for the number of predictors in the regressions with a desired statistical power of 0.95 and a probability level of 0.01.
iii) Regarding modelling methods, we controlled for alternate modelling approaches, such as a single regression model including all the variables. The single model provided comparable results but was much less practical to analyse, given the potential risks of endogeneity and collinearity between the numerous variables. We performed successful post-estimation tests for each of the three models presented above. There was no misspecification error. Each model appeared to be well-defined, meaning there were no signs of omitted variables. The goodness-of-fit test indicated that each model fits the data well. Including interactions improved the model, and finally, we detected no sign of collinearity. The complete test results are presented in the supplementary material.

## Discussion

Over the past two and a half years, the COVID-19 pandemic and its social and economic effects have disrupted health and HIV services. HIV key population groups such as transgender people are at greater risk of HIV vulnerability.

This cross-sectional survey provides some preliminary insights into how the COVID-19 pandemic may have exacerbated pre-existing vulnerability among women and girls at high risk of HIV and those living with HIV. To the best of our knowledge, this is the first study looking at socioeconomic inequality, health inequity and the well-being of transgender people during COVID-19 in Africa. The study indicated that differences in the COVID-19 pandemic induced socioeconomic inequality between cisgender women and transgender people, with transgender people being worse off socio-economically and more likely to have to deal with housing insecurity. Also, almost a third of transgender people had challenges accessing gender-affirmative treatment and were more likely to have difficulties accessing HIV services. Transgender people were also more likely to experience gender-based violence, and moderate to severe anxiety and depression during the pandemic.

One of the strengths of the current study is the community representatives’ active engagement in the survey design and implementation. It contributed to reaching out to vulnerable and marginalised population groups, particularly remote territories, during COVID-19. Another strength of the study is the large sample of participants among these two vulnerable gender categories, enabling potent statistical analyses. The study additionally provides evidence of the multidimensional vulnerabilities of transgender during external shocks such as the one induced by the COVID-19 health crisis.

The study, however, has some limitations. First, there is a risk of study participants’ selection bias inherent to convenience sampling techniques. Using non-probability sampling techniques is generally the most appropriate method for recruiting hard-to-reach and stigmatised populations such as the ones for this study. Using multiple non-probability sampling techniques, such as the venue-based and snowball sampling methods, helps to ensure the diversity of study participants [62]. Additional participants were recruited through the river sampling method. Second, the study measures were self-reported, which may increase the risk of over- or under-estimation. We used validated instruments to help minimise this risk. These limitations acknowledged, the study identified several relevant points.

First, regarding socioeconomic inequality, though both cisgender women and transgender participants had comparable subjective social standing and financial vulnerability during the COVID-19 pandemic, transgender people seemed to face socioeconomic inequality. Despite reporting higher education achievements and good health, transgender participants were one and a half more likely to be in the lower subjective socioeconomic tercile than vulnerable cisgender women and have a higher risk for housing insecurity. Stigma and discrimination create or exacerbate housing instability for transgender people, which in turn may increase sexual risk behaviour [63–65]. There are no prior reports of a housing crisis among transgender people in Nigeria; however, the “invisibility” of transgender people and poor reporting of transgender issues in Nigeria may have created a gap. Possible mediating factors that may increase the risk of transgender people to housing insecurity include residency in urban areas [66]. It is, however, described elsewhere that housing insecurity may be a manifestation of stigma and further trigger anxiety and depression in transgender people [67]. Gender-based violence has a further disproportional impact on the mental health of transgender people [68–70]. The current study indicates that transgender people were more likely to have experienced gender-based violence during the COVID-19 pandemic. In addition to the threat of the pandemic to physical health, mental health is further challenged by the emotional response to the situation and the official imposed public health measures [71, 72]. As the study results have reflected, the COVID-19 pandemic may have triggered new factors that negatively impact the life of transgender individuals in Nigeria. Further research is required to assess these findings.

Second, in terms of health inequity, the study found that the crude self-reported HIV-positive status was higher among transgender participants than among key and vulnerable cisgender women and girls. Transgender people were nearly three times more likely to engage in sex work and transactional sex than vulnerable cisgender women. Nevertheless, transgender participants were more likely to have performed a recent HIV test and were more than half less likely to have never been tested for HIV. Moreover, the study also found that among those who engaged in sex work, transgender participants reported a comparable, if not better, use of HIV and STI prevention methods during the COVID-19 crisis in Nigeria. These findings suggest that transgender people could be aware of their HIV risks and engage more in HIV and STI prevention measures. This point is of particular interest and opens the door to further research to explore how the transgender community and community-based organisations serving the needs of community members organised themselves during the pandemic to play a substantial role in promoting access to HIV and STI prevention methods.

The difference in financial barriers to accessing healthcare services between transgender and vulnerable cisgender women probably deserves more attention. On the one hand, cisgender women identified the standard or regular fees at the health centre as a financial barrier, not the non-official fees. On the other hand, transgender people reported non-official fees as the primary financial barrier; the ratio of probabilities for the standard fees was not statistically different from those faced by cisgender women. This finding highlights the significant role of non-official fees as a barrier to accessing health and HIV services for transgender people. The results partially supported the study hypotheses, showing an increased economic vulnerability and health inequity of transgender people compared to vulnerable cisgender women living with or at high risk of HIV. The study also identified the concern for disclosure of gender identity and sexual orientation as a significant non-financial structural barrier to accessing healthcare. These findings emphasise how the absence of basic human rights and being outlawed affects access to health and health outcomes in key and vulnerable groups such as transgender people. This has also been described elsewhere [73].

Only about a quarter of the transgender participants reported having access to gender-affirmative commodities and disrupted access to gender-affirmative services. Similar findings were described in other countries [74–77]. The study did not explore how transgender individuals addressed this challenge. Communities of transgender people in other parts of the world found alternative sources of commodity supplies and service access through telehealth [78]. Telehealth also facilitated access to providers with the competency to address the physical and mental health needs of transgender populations [79]. Future studies could explore how community members negotiated these community-specific health care need challenges; and the implication of instituting practices that can enhance access to transgender community-specific services during crisis periods.

Third, the findings highlighted acute concerns regarding mental health and the well-being of transgender people. Discrimination, abuse, harassment, and violence are common experiences for transgender people. From a young age, they often face stigma, discrimination and social rejection in their homes and communities for expressing their gender identity. Our study found that transgender people are more than twice and half more likely to suffer gender-based violence than cisgender vulnerable women. While two third of vulnerable cisgender women reported not experiencing violence, only 43% of transgender participants reported not experiencing violence. More worrying is that one in ten vulnerable cisgender women reported increased gender-based violence since the COVID-19 crisis started, while one in five transgender people did. This essential finding points to the need for more coordinated interventions tackling the issue of gender-based violence in key and vulnerable populations.

We also found a significant difference in mental health between cisgender women and transgender participants. A third of vulnerable cisgender women showed moderate to severe symptoms of anxiety and depression, while nearly half of transgender people showed moderate to severe symptoms of anxiety and depression. Other studies confirm this [80].

Finally, the study included three validated psychoeconomic decision-making preferences and found that transgender participants were more likely to take risks than vulnerable cisgender women. Their mean score was 27% higher than vulnerable cisgender women. Transgender participants also had lower time preferences. In other terms, transgender participants were more likely than vulnerable cisgender women to discount future outcomes when comparing them with more immediate outcomes. Several research projects have elicited the relationship between time preference and health risk behaviour [81, 82] and preventive health behaviour [83], including HIV [58, 84–86]. These findings bring new dimensions to prior studies focusing on the high sexual risk-taking behaviour of transgender people [87]. There may be differences in contextual factors driving high risk-taking behaviour and health care avoidance [88].

## Conclusion

This study brings to light new dimensions of the vulnerability of transgender people in a highly stigmatising sub-Saharan country. It identified how public health crises like the COVID-19 pandemic could exacerbate socioeconomic inequality, health inequity, and well-being. By comparing key and vulnerable cisgender women and transgender people in Nigeria, the study highlighted how the alarming situation of key and vulnerable women in some African countries is worse for transgender people. There is a possibility that the increased vulnerability of vulnerable cisgender women and transgender people induced by the COVID-19 pandemic will turn into long-term public health consequences that jeopardise the achievements of the Sustainable Development Goals. While further studies are suited to explore the medium and long-term impact of the health shock created by the COVID-19 pandemic on transgender people in Africa, civil society organisations, national programmes and the donor community should take stock of the new evidence revealed by this study and implement interventions to address the socioeconomic inequality, health inequity and well-being of transgender people. This includes introducing procedures and legislations that protect key and vulnerable population groups such as transgender people from stigma, discrimination, and violence, including in health care facilities.

### Supplementary Information

**Additional file 1: Supplement S1.** Terciles of the Subjective Social and Socioeconomic Status. **Supplement S2.** Questonnaire (English version)

## Data Availability

The data that support the findings will be available from the corresponding author upon request following a 6-month embargo from the date of publication. Requests will be controlled, and each request will be considered on a case-by-case basis.

## Abbreviations

aOR: Adjusted Odds Ratio
CI: Confidence Interval
COVID-19: Coronavirus Infectious Disease 2019
HIV: Human Immunodeficiency Virus
SES: Subjective socioeconomic status
SSS: Subjective social standing status
