# Supplementary material for: Socioeconomic inequality, health inequity and well-being of transgender people during the COVID-19 pandemic in Nigeria
Source: BMC Public Health. 2023 Aug 12;23:1539. doi: 10.1186/s12889-023-16482-1 (PMC10422710; doi:10.1186/s12889-023-16482-1)
Supplement: Supplementary file 1 — Additional file 1: Supplement S1. Terciles of the Subjective Social and Socioeconomic Status. Supplement S2. Questonnaire (English version) [file 12889_2023_16482_MOESM1_ESM.pdf]

Supplement S1: Terciles of the Subjective Social and Socioeconomic Status

Subjective socioeconomic status was assessed using the McArthur scale [36] with the following question: "Think of a ladder representing where people stand in your country. At the top of the ladder are the people who are the best off. At the bottom are the people who are the worst off. Where would you place yourself on this ladder at this moment?" Possible answers were a 10-item Likert scale ranging from “1: among those having the least money, least education and least respected jobs or no job”, to “10 among those having the most money, most education and most respected jobs”. The participants' responses were grouped per tercile.

Table 1: participants per subjective socioeconomic status

SES scale	Number	Percent	Cumulative	Tercile
1	976	22.01	22.01	1
2	634	14.3	36.31	1
3	706	15.92	52.23	2
4	573	12.92	65.16	2
5	611	13.78	78.94	2
6	336	7.58	86.51	3
7	241	5.44	91.95	3
8	155	3.5	95.44	3
9	89	2.01	97.45	3
10	113	2.55	100	3

Subjective social status was assessed using the McArthur scale [36] with the following question: "Now, think of a ladder representing where people stand in your local community. Where would you place yourself on this ladder at this moment?" Possible answers were a 10-item Likert scale ranging from “1: lowest standing in my community”, to “10: highest standing in my community”. The participants' responses were grouped per tercile.

Table 2: Participants per subjective social status

SSS scale	Number	Percent	Cumulative	Tercile
1	808	18.19	18.19	1
2	566	12.74	30.93	1
3	702	15.8	46.73	1
4	583	13.12	59.85	2
5	697	15.69	75.53	2
6	424	9.54	85.08	3
7	276	6.21	91.29	3
8	193	4.34	95.63	3
9	78	1.76	97.39	3
10	116	2.61	100	3

Supplement S2: Questionnaire (English version)

Nigeria 64

Survey on the impact of COVID-19 on HIV services, wellbeing, and economic vulnerability of women and girls living with or at risk for HIV

Dear Participant,

Thank you for participating in this survey which will help improve actions taken in response to the COVID-19 pandemic and inform the response to similar future outbreaks. The purpose of this survey is to understand and explore the various impacts of the COVID-19 crisis on the health and wellbeing of women and girls living with or at risk for HIV in Nigeria, as well as related economic shocks as a driver of heightened vulnerability of women and girls.

This study will involve completing a 20-minute survey that will ask you questions relating to the coronavirus.

Participation is open to woman and girls aged at least 15-year-old, living in Nigeria and is voluntary. This survey is anonymous. Please be reassured, that all responses are confidential. You can skip question you are not comfortable with. You can also stop participating to the survey at any moment by closing the page.

This study is conducted by the Institute of Public Health, Obafemi Awolowo University, in collaboration with UNAIDS. It has been approved by the Ethics Review Committee of the Institute of Public health, Obafemi Awolowo University, Ile-Ife, Osun State, Nigeria (approval number: IPHOAU/12/1692).

By taking part, you are agreeing that you have read and understood the information about the study provided below. Please click on "show policy" below for more details about the study, and contact details of the research group.

There are 145 questions in this survey.

Socio-demographic background

Please tell us a bit about you and your background.

All your answers are confidential and nobody can identify you. It is anonymous.

1 Data Collector's Identification 64

2
In which geopolitical zone do you currently live?*

Please choose **only one** of the following:

- ◯ North Central
- ◯ North East
- ◯ North West
- ◯ South East
- ◯ South South
- ◯ South West

3 How old are you?* *

Please choose **only one** of the following:

- ◯ Under 15
- ◯ 15-18
- ◯ 19-24
- ◯ 25-30
- ◯ 31-34
- ◯ 35-39
- ◯ 40-44
- ◯ 45-49
- ◯ 50-54
- ◯ 55-59
- ◯ 60-64
- ◯ 65 +

4 Can you confirm your ethnicity?

Please choose **only one** of the following:

- ◯ Birom
- ◯ Bura
- ◯ Edo
- ◯ Efik
- ◯ Fulani
- ◯ Gwari
- ◯ Hausa
- ◯ Ibibio
- ◯ Idoma
- ◯ Igala
- ◯ Igbo
- ◯ Ijaw
- ◯ Ikwere
- ◯ Itsekiri
- ◯ Kaje
- ◯ Kanuri
- ◯ Okrika
- ◯ Nupe
- ◯ Shuwa-Arab
- ◯ Urhobo
- ◯ Tiv
- ◯ Yoruba
- ◯ I cannot or do not wish to answer this
- ◯ I don't know
- ◯ Other ____________

5 What is your religion?

Please choose **only one** of the following:

- Catholic
- Hinduism
- Islam
- Judaism
- Protestant
- Traditional
- None
- I prefer not to answer
- Other ____________

6 Do you identify having a disability (do you have a physical or mental condition that limits your movements, senses, or daily activities)?

Please choose **only one** of the following:

- Yes
- No
- I cannot or do not wish to answer this question
- I don't know

7 Do you have difficulty in the following domains?

Please choose the appropriate response for each item:

	No difficulty	Some difficulty	A lot of difficulty	Cannot function at all
**Seeing even if wearing glasses**	○	○	○	○
**Hearing even if using a hearing aid**	○	○	○	○
**Walking or climbing steps**	○	○	○	○
**Remembering or concentrating**	○	○	○	○
**Speech (like using your usual language)**	○	○	○	○
**Personal care (like washing all over or dressing)**	○	○	○	○

8 What is your highest level of schooling completed?

Please choose **only one** of the following:

- ○ No formal education
- ○ Quranic only
- ○ Primary school
- ○ High school or secondary school
- ○ Trade school, vocational training, or apprenticeship
- ○ Postsecondary education or some college
- ○ University

9 What type of dwelling or house do you live in?

Please choose **only one** of the following:

- ○ A house or a flat
- ○ A traditional house like a mud hut
- ○ An informal house like a shack
- ○ A refugee camp / temporary shelter
- ○ A women’s shelter
- ○ Rehabilitation centre
- ○ I have no place to live in
- ○ Other ______

10 In your household, is there*:*

Please choose **all** that apply:

- ☐ Electricity
- ☐ A mobile phone
- ☐ A television
- ☐ A gas or electric stove
- ☐ A refrigerator
- ☐ A computer
- ☐ Internet at home
- ☐ A bicycle
- ☐ A motorcycle
- ☐ A car
- ☐ Does not own any of the items on this list

11 What is the main source of drinking water for members of your household?

Please choose **only one** of the following:

- ◯ Piped water into dwelling
- ◯ Piped water into yard
- ◯ Piped to neighbour
- ◯ Public tap / standpipe
- ◯ Tube well or borehole
- ◯ Dug well, unprotected
- ◯ Dug well, protected (wellhead is protected by a plastered cement slab)
- ◯ Water from spring
- ◯ Rainwater
- ◯ Tanker truck
- ◯ Cart with small tank
- ◯ Surface water (river, dam, lake, canal, ...)
- ◯ Bottled water
- ◯ Other ____________

12 What kind of toilet facility do members of your household usually use?

Please choose **only one** of the following:

- ◯ Flush toilet
- ◯ Pit latrine with slab
- ◯ Pit latrine without slab / open pit
- ◯ No facility / bush /field

13 Does any members of this household own any agricultural land?

Please choose **only one** of the following:

- ◯ Yes
- ◯ No

14 Which of the following describes the place where you now live?

Please choose **only one** of the following:

- ◯ A large city
- ◯ A town
- ◯ A village
- ◯ A farm or isolated house

15 In this city, you live in:

Please choose **only one** of the following:

- ◯ A wealthy suburb
- ◯ A middle-class suburb
- ◯ A poor suburb
- ◯ A slum
- ◯ A refugee camp / temporary shelter
- ◯ I cannot or do not wish to answer this question

16 Indicate total number of people living with you in your household?

Please choose **only one** of the following:

- ◯ 1 (only me)
- ◯ 2
- ◯ 3
- ◯ 4
- ◯ 5
- ◯ 6
- ◯ 7
- ◯ 8
- ◯ 9
- ◯ 10
- ◯ 11
- ◯ 12
- ◯ 13
- ◯ 14
- ◯ 15 and more

17 With whom do you currently live?

Please choose **all** that apply:

- ☐ Alone, no other person
- ☐ Spouse
- ☐ A sexual or romantic partner (non-spouse)
- ☐ Other adult family members
- ☐ Close friend(s) or roommate(s)
- ☐ Children under the age of 18
- ☐ Children above the age of 18
- ☐ Any other adult (not close friend)
- ☐ Other

18 What is your current relationship status?

Please choose **all** that apply:

- [ ] Single
- [ ] Married
- [ ] In a relationship but not living together
- [ ] In more than one relationship
- [ ] Divorced / separated
- [ ] Widowed
- [ ] Other
- [ ] I cannot or do not wish to answer this question

19 How many children do you have?

Please choose **only one** of the following:

- ○ 0
- ○ 1
- ○ 2
- ○ 3
- ○ 4
- ○ 5
- ○ 6
- ○ 7
- ○ 8
- ○ 9
- ○ 10 or more

20 Do you have young children (under 18y)?

Please choose **all** that apply:

- ☐ Yes, living with me
- ☐ Yes, but not living with me
- ☐ No, my children are above 18
- ☐ I don't know
- ☐ I cannot or do not wish to answer this question

21 Who do you have sex with?

Please choose **only one** of the following:

- ○ Men only
- ○ Women only
- ○ Both men and women
- ○ Other (example: asexual, pansexual, …)
- ○ I have never had sex
- ○ I don't know
- ○ I cannot or do not wish to answer this question

22 How would you primarily define your gender identity?

Please choose **only one** of the following:

- ○ Woman
- ○ Transgender woman (male to female)
- ○ Transgender man (female to male)
- ○ I don't know
- ○ I cannot or do not wish to answer this question
- ○ Other ________________

23 What sex were you assigned at birth?

Please choose **only one** of the following:

- ◯ Female
- ◯ Male
- ◯ Intersex - my sex was unclear at birth and/or I was diagnosed with an intersex condition

The COVID-19 epidemic

This section contains questions on the health and non-health impacts of the COVID-19 pandemic on you.

24 In general, how would you rate your health?

Please choose **only one** of the following:

- ◯ Excellent
- ◯ Very good
- ◯ Good
- ◯ Fair
- ◯ Poor

25 Do you use public transport?

Please choose **only one** of the following:

- ◯ Yes, more than before the COVID-19 crisis
- ◯ Yes, same as before the COVID-19 crisis
- ◯ Yes, less than before the COVID-19 crisis
- ◯ Not anymore since COVID-19 crisis
- ◯ No, never

26 Have you or anyone in your household been screened or tested for COVID-19 ?

Please choose **only one** of the following:

- ◯ Yes
- ◯ No
- ◯ I don't know

27 Have you or anyone in your household ever had a positive COVID-19 test result?

Please choose **only one** of the following:

- ◯ Yes
- ◯ No
- ◯ I don't know

28 What precautions do you take to avoid becoming infected or reinfected with COVID-19?

Please choose **all** that apply:

- ☐ No particular measure
- ☐ Wash or sanitize my hands frequently
- ☐ Wearing a re-usable cloth or disposable face mask
- ☐ Avoid crowded areas
- ☐ Avoid going out
- ☐ Daily monitoring of signs and symptoms
- ☐ Going to a health facility or testing site for a COVID-19 test if I have signs or symptoms
- ☐ Getting vaccinated

29 What challenge do you face in taking measures to prevent COVID-19 infection?

Please choose **all** that apply:

- ☐ There is no place to wash my hands around where I live
- ☐ I cannot afford to pay for cloth or disposable face masks
- ☐ I cannot afford to buy soap or sanitizers
- ☐ I cannot avoid crowded areas
- ☐ I already had COVID-19
- ☐ I don’t really care about COVID-19
- ☐ There is no COVID-19 here
- ☐ Other

30 Are you worried that you or someone in your immediate family may become seriously ill from COVID-19?

Please choose **only one** of the following:

- ○ Very worried
- ○ Somewhat worried
- ○ Not too worried
- ○ Not worried at all

31 What makes you confident that you or your relative will not become seriously ill from COVID-19?

Please choose **all** that apply:

- [ ] I follow preventive measures (handwashing, physical distancing, masks, etc.)
- [ ] We are strong and healthy
- [ ] Weather is hot here
- [ ] God will protect us
- [ ] It is curable/treatable
- [ ] There is no COVID-19 here
- [ ] Other: ____________

32 When a vaccine for COVID-19 is available to me, I will get it

Please choose **only one** of the following:

- ( ) Strongly agree
- ( ) Agree
- ( ) Disagree
- ( ) Strongly disagree

33 What are the reasons you are not sure you want a vaccine for COVID-19?

Please choose **all** that apply:

- ☐ I think the risk to me of getting COVID-19 is low
- ☐ I'm against vaccines in general
- ☐ It is not a priority for me
- ☐ I am afraid of side effects
- ☐ My family or the person I live with (husband, partner, etc) is against it
- ☐ Registering for it could put me in trouble due to my situation (example I'm an illegal worker, migrant, etc)
- ☐ I think it is against my religion
- ☐ I do not trust what the authorities say
- ☐ I do not have time to go to a health centre for a vaccine
- ☐
- ☐ Other: ____________

34 Did you have to stop school/ university/ college due to the COVID-19 pandemic?

Please choose **only one** of the following:

- ○ Yes
- ○ No
- ○ Does not apply to me

35 What made you stop school during COVID-19 pandemic?

Please choose **all** that apply:

- [ ] The school/ university/ college was closed due to the COVID-19
- [ ] My parents or my family did not allow this
- [ ] Nobody to look after my child/children
- [ ] Money problems
- [ ] Stigma
- [ ] I had no data /internet to attend online classes
- [ ] I became pregnant
- [ ] I don't know
- [ ] Other

36 Are you planning to return to school after the pandemic?

Please choose **only one** of the following:

- ○ Yes, I have already returned to school
- ○ Yes, later
- ○ No
- ○ I don’t know

Your economic and social situation

This page includes questions on work, income, benefits, and how COVID-19 crisis affected your situation.

37 How many people contribute financially to your household (you included)?

Please choose **only one** of the following:

- ◯ 1 (only me)
- ◯ 2
- ◯ 3
- ◯ 4
- ◯ 5
- ◯ 6
- ◯ 7
- ◯ 8
- ◯ 9
- ◯ 10 and more

38
In the last 30 days, did you do any work, do any kind of business, farming, or other activity in exchange of a salary, cash or in kind, even if only for few hours?

Please choose **only one** of the following:

- ◯ Yes
- ◯ No

39 Are you paid in cash or in-kind for this work or are you not paid at all?

Please choose **only one** of the following:

- ◯ Cash only
- ◯ Cash and kind
- ◯ In kind only
- ◯ Not paid

40 What is the main reason you are not currently working?

Please choose **only one** of the following:

- ◯ Business or office closed due to Covid legal restrictions
- ◯ Business or office closed for another reason
- ◯ I was retrenched or lost my job
- ◯ I have been temporarily told not to work
- ◯ I am ill or quarantined
- ◯ I need to care for the children while schools are closed
- ◯ I need to care for an ill relative
- ◯ Not able to work or farm due to lack of inputs
- ◯ It is not the farming season
- ◯ I'm a student
- ◯ I'm pregnant or on maternity
- ◯ I am unable to work due to a disability
- ◯ I am retired or I am too old
- ◯ Other

41 Currently, what are your main sources of income? (maximum 3)

Please choose **all** that apply:

- ☐ I have paid work
- ☐ I am self-employed/ have my own business (example: hairdresser or sex worker)
- ☐ I am a daily wage earner (example: domestic workers)
- ☐ Petty trade
- ☐ From agriculture
- ☐ I am in survival mode (example: recycling and selling in slums, begging)
- ☐ Money sent by family members (inside or outside of Nigeria)
- ☐ Assistance from the Government (social grants or the COVID-19 grant)
- ☐ Assistance from NGOs / charitable organisation
- ☐ Pension for me or a family member
- ☐ I am engaging in transactional sex or I have a sugar daddy
- ☐ I do not have any source of income
- ☐ Other: ____________

Select the bigger source of income. If needed you may chose a maximum of 3 sources of income

42 Did your total income change since COVID-19 started?

Please choose **only one** of the following:

- ○ Increased
- ○ No change
- ○ Decreased

43 How much has your income been reduced due to the COVID-19 crisis?

Please choose **only one** of the following:

- ◯ No change in my income
- ◯ reduced by less than half my income
- ◯ reduced by about half my income
- ◯ reduced by more than half my income
- ◯ I lost all my income

44 How are you coping with this reduction in your income?

Please choose **all** that apply:

- ☐ Relied on savings
- ☐ Reduced food consumption
- ☐ Reduced non-food consumption
- ☐ Did nothing
- ☐ Transactional sex or Sex work
- ☐ Received assistance
- ☐ Help from family
- ☐ Sale of assets
- ☐ Other: ____________

45 Do you access special COVID-19 relief grant or other COVID-19 support measures (example: social grants or food vouchers)

Please choose **only one** of the following:

- ◯ Yes I applied, and I received these support measures
- ◯ Yes I applied, and I am waiting for the support measure
- ◯ I can access these support measures if I want, but I don’t
- ◯ I have been denied access
- ◯ These measures are not applicable to me
- ◯ I did not know there was special relief measure for me
- ◯ I cannot or do not wish to answer this question

46 Since the COVID-19 crisis began, do you eat less or skip meals because there was not enough money for food?

Please choose **only one** of the following:

- ◯ Yes
- ◯ No

47 Do you have enough money today to cover the daily expenses of today and tomorrow?

Please choose **only one** of the following:

- ◯ Yes
- ◯ No

48 Do you have a bank account in your name?

Please choose **only one** of the following:

- Yes
- No

49
Do you currently participate in a Esusu, Adashe, Ajo or other cooperative?

Please choose **only one** of the following:

- Yes
- No

50 In the past 6 months, did you not pay or underpay your rent or bond?

Please choose **only one** of the following:

- Yes
- No
- I cannot or do not wish to answer this question
- Does not apply to me

51 Since the COVID-19 crisis began, did you move in with other people, even for little while, because of financial problems?

Please choose **only one** of the following:

- ◯ Yes
- ◯ No
- ◯ I cannot or do not wish to answer this question

52 How many times did you have to move since COVID-19 crisis began?

Please choose **only one** of the following:

- ◯ 0
- ◯ 1
- ◯ 2
- ◯ 3
- ◯ 4
- ◯ 5
- ◯ more than 5
- ◯

53

Think of a ladder representing where people stand in your country.

At the top of the ladder are the people who are the best off. At the bottom are the people who are the worst off.

Where would you place yourself on this ladder at this moment?

Please choose **only one** of the following:

- ◯ 10, among those having most money, most education and most respected jobs
- ◯ 9
- ◯ 8
- ◯ 7
- ◯ 6
- ◯ 5
- ◯ 4
- ◯ 3
- ◯ 2
- ◯ 1, among those having the least money, least education and least respected jobs or no job

54
Now, think of a ladder representing where people stand <u>in your local community</u>.

Where would you place yourself on this ladder at this moment?

Please choose **only one** of the following:

- ◯ 10, highest standing in my community
- ◯ 9
- ◯ 8
- ◯ 7
- ◯ 6
- ◯ 5
- ◯ 4
- ◯ 3
- ◯ 2
- ◯ 1, lowest standing in my community

Behavioural economics

This section is on how you make your choices and your decisions, how you decide courses of action.

55 How willing are you to take risks, in general?

Please choose **only one** of the following:

- ◯ 10, very willing
- ◯ 9
- ◯ 8
- ◯ 7
- ◯ 6
- ◯ 5
- ◯ 4
- ◯ 3
- ◯ 2
- ◯ 1
- ◯ 0, very unwilling

56 How willing are you to give to good causes without expecting anything in return?

Please choose **only one** of the following:

- ◯ 10, very willing to do so
- ◯ 9
- ◯ 8
- ◯ 7
- ◯ 6
- ◯ 5
- ◯ 4
- ◯ 3
- ◯ 2
- ◯ 1
- ◯ 0, very unwilling to do so

57
Imagine the following situation: Today you unexpectedly received 20,000 Naira. How much of this amount would you donate to a good cause?

Please write your answer here:

58 How willing are you to give up something that is beneficial for you today in order to benefit more from that in the future?

Please choose **only one** of the following:

- 10, very willing to do so
- 9
- 8
- 7
- 6
- 5
- 4
- 3
- 2
- 1
- 0, very unwilling to do so

59 How well does the following statement describe you as a person: “I only act to satisfy immediate concerns, figuring that I will take care of future problems that may occur at a later date”

Please choose **only one** of the following:

- ◯ 5, it describes me perfectly
- ◯ 4, it somewhat describes me
- ◯ 3, uncertain
- ◯ 2, it somewhat does not describe me
- ◯ 1, it does not describe me at all

60 How well does the following statement describe you as a person: “I assume that people have only the best intentions”

Please choose **only one** of the following:

- ◯ 10, describes me perfectly
- ◯ 9
- ◯ 8
- ◯ 7
- ◯ 6
- ◯ 5
- ◯ 4
- ◯ 3
- ◯ 2
- ◯ 1
- ◯ 0, does not describe me at all

Your health and wellbeing

This section contains questions on health and non-health impacts of the COVID-19 pandemic.

Remember, your answers are confidential and you may skip a question if you are not

comfortable with it.

61 Imagine a ladder with steps representing happiness in life, with the 10th step being the happiest. On which step of the ladder do you stand at this time?

Please choose **only one** of the following:

- ◯ 10, the best possible life for me
- ◯ 9
- ◯ 8
- ◯ 7
- ◯ 6
- ◯ 5
- ◯ 4
- ◯ 3
- ◯ 2
- ◯ 1
- ◯ 0, the worst possible life for me

62 Over the past 2 weeks, how often have you been bothered by the following problems?

Please choose the appropriate response for each item:

	Nearly every day (12-14 days)	More than half the days (8-11 days)	Several days (1-7 days)	Not at all (0 day)
**Feeling down, depressed or hopeless**	○	○	○	○
**Little interest or pleasure in doing things**	○	○	○	○
**Feeling nervous, anxious or on edge**	○	○	○	○
**Not being able to stop or control worrying**	○	○	○	○

63 In the past 6 months, how often have you thought about taking your own life?

Please choose **only one** of the following:

- ○ Never
- ○ Seldom
- ○ Very often
- ○ All the time

64
If you're thinking about suicide, be sure to talk to other people about it. They can be friends or family members, but they don't have to be. It can be difficult to talk about this topic with people close to you. What is important is that you talk to someone.

You can do this by phone, chat, e-mail or in person Nigeria Suicide Hotline:

+234 (0) 806 210 6493

+234 (0) 809 210 6493

65 Which of the following healthcare services did you access in the last year?

Please choose **all** that apply:

- ☐ Public Healthcare (clinic, hospital)
- ☐ Private Healthcare (clinic, hospital, pharmacy)
- ☐ Traditional healer
- ☐ None, I don't want to use any healthcare services
- ☐ I cannot afford any healthcare
- ☐ Other: ____________

66

Did COVID-19 pandemic have an impact on your attendance at the health facilities for the following services when you needed them?

Please choose the appropriate response for each item:

	Yes	No	Not needed
HIV services	◯	◯	◯
Tuberculosis services	◯	◯	◯
Family planning	◯	◯	◯
Safe abortion care	◯	◯	◯
Sexually transmitted infection (STI)	◯	◯	◯
Services for gender-based violence or intimate partner violence	◯	◯	◯
Gender-affirming hormone therapy	◯	◯	◯
Other chronic care (diabetes, hypertension, cardiovascular disease)	◯	◯	◯

67 Are there costs which are preventing you from accessing the HIV or health services you needed above?

Please choose **all** that apply:

- ☐ Transportation cost
- ☐ The cost of medicines or tests
- ☐ Fees at the clinic or hospital
- ☐ The additional nonofficial fees (example baksheesh)
- ☐ The income I would miss out on while going to access these health services
- ☐ I have no money or all the money I have is being used for food and essential items
- ☐ I prefer not to answer
- ☐ None, I have no financial issues to access healthcare services
- ☐ Other: ________

68 Are there other reasons that prevent you from accessing family planning, or the HIV services you needed above?

Please choose **all** that apply:

- ☐ The road to the health facility is risky for me (e.g. violence, high risk of sexual assault)
- ☐ I'm worried people could discover my sexual orientation
- ☐ I'm worried people could discover my HIV status
- ☐ Last time I went for HIV or health service I was humiliated
- ☐ Last time I went for HIV or health service I faced improper treatment (violence, insult, or discrimination)
- ☐ I do not have time to go to the health facility
- ☐ The service was offered at school or at a local NGO and it is now closed due to COVID-19
- ☐ I am not ready to deal with my HIV infection
- ☐ I fear being infected with COVID-19 in the health facility
- ☐ I prefer not to answer
- ☐ Other: ________

69 Did you become pregnant during the COVID-19 pandemic?

Please choose **only one** of the following:

- ◯ Yes
- ◯ No
- ◯ I don't know
- ◯ I cannot or do not wish to answer

70 Was this pregnancy planned?

Please choose **only one** of the following:

- ◯ Yes
- ◯ No
- ◯ No, I was raped
- ◯ I don't know
- ◯ I cannot or do not wish to answer this question

71
If you are a survivor of rape, we encourage you to report the incident to the nearest police station and health facility.

Please do not hesitate to reach out for assistance. Take a screenshot of these numbers:

Hotline: 08052004698 and 08180091072

Helpline for child abuse: 08085753932 or 08102678442

Website: http://www.projectalertnig.org (http://www.projectalertnig.org/)
Facebook page: http://www.facebook.com/ProjectAlertOnViolenceAgainstWomen (https://www.facebook.com/ProjectAlertOnViolenceAgainstWomen/)

72 Have you attended antenatal care during this pregnancy?

Please choose **only one** of the following:

- ◯ Yes
- ◯ No
- ◯ I don't know
- ◯ I cannot or do not wish to answer

73 Where did/do you plan on delivering your baby?

Please choose **only one** of the following:

- ◯ Health facility
- ◯ Home with assistance of community health worker
- ◯ Home, non-assisted
- ◯ I don't know

74 When was your last HIV test? *

Please choose **only one** of the following:

- ◯ In the last 3 months
- ◯ 4 to 6 months ago
- ◯ 7 to 12 months ago
- ◯ More than 12 months ago
- ◯ I never had an HIV test

75 Do you know your HIV status?* *

Please choose **only one** of the following:

- ◯ I am HIV-positive
- ◯ I am HIV-negative
- ◯ I don't know
- ◯ I cannot or do not wish to answer

76 Are you currently taking HIV (antiretroviral) treatment?

Please choose **only one** of the following:

- ◯ Yes
- ◯ No

77 Is there a reason you are not taking HIV antiretroviral therapy?

Please choose **all** that apply:

- ☐ I used to, but stopped since COVID-19 crisis started
- ☐ Medication is currently not available at the clinic or hospital
- ☐ I am unable to collect medications at the clinic or hospital
- ☐ I cannot tolerate medication side effects or am worried about taking the pills
- ☐ I do not feel treatment is needed
- ☐ I am worried someone would find out my HIV status
- ☐ I am not ready to deal with my HIV infection
- ☐ I am worried the healthcare workers would treat me badly or disclose my HIV status without my consent
- ☐ I do not qualify for treatment because my CD4s are too high
- ☐ I am not sure I can access it as migrant or refugee
- ☐ No, for other reasons

78 Since COVID-19 crisis started, did you have an HIV viral load or CD4 test?

Please choose **only one** of the following:

- ◯ Yes
- ◯ No
- ◯ I don't know

79 What are the reasons you could not have an HIV viral load of CD4 test in the last 12 months?

Please choose **only one** of the following:

- It was planned and cancelled because of COVID-19
- I cannot afford the costs of the test
- I did not follow up with my lab test
- I was not aware I should do regular HIV viral load test every year
- I am not sure I can do HIV viral load test here as migrant or refugee
- I don't know what "viral load" and "CD4 test" mean

Gender-based violence

Since COVID-19 crisis started, many girls and women have been facing increased violence. This violence can be physical or sexual, but it can also be emotional and financial.

This section poses sensitive questions. You may skip any question you are not comfortable answering.

80 Do you feel that you are currently experiencing:

Please choose **only one** of the following:

- More violence than before the COVID-19 crisis
- The same level of violence as before the COVID-19 crisis
- Less violence than before the COVID-19 crisis
- I am not experiencing any violence
- I cannot or do not wish to answer this question

81 From whom are you experiencing violence?

Please choose **all** that apply:

- ☐ Family
- ☐ Romantic / Sexual partner
- ☐ Friends
- ☐ Neighbours
- ☐ Healthcare providers
- ☐ Police and/or military
- ☐ Employer or co-workers
- ☐ Religious or faith community
- ☐ Teachers or school
- ☐ Government representatives
- ☐ Reporters and the media
- ☐ Other

82 Since COVID-19 crisis began, how often did your partner, husband or parent try to keep you from going to work or school?

Please choose **only one** of the following:

- ○ Very often
- ○ Often
- ○ Sometimes
- ○ Rarely
- ○ Never

83 Since the COVID-19 crisis began, how often did your partner, husband or parent withhold money, make you ask for money, or take your money?

Please choose **only one** of the following:

- Very often
- Often
- Sometimes
- Rarely
- Never

84 Since COVID-19 crisis began, how often did your partner, husband or parent try to keep you from seeing friends and family?

Please choose **only one** of the following:

- Very often
- Often
- Sometimes
- Rarely
- Never

85 Since COVID-19 crisis began, how often did your partner, husband or parent try to insult and criticize you?

Please choose **only one** of the following:

- Very often
- Often
- Sometimes
- Rarely
- Never

86 Since the COVID-19 crisis began, has your partner/s or husband slapped, punched, kicked, or shoved you?

Please choose **only one** of the following:

- Yes
- No
- I cannot or do not wish to answer this question

87 If yes, did this happen...

Please choose **only one** of the following:

- Before the COVID-19 crisis
- Since the COVID-19 crisis started
- Both before and after the COVID-19 crisis

88 How often did this happen since the COVID-19 crisis began?

Please choose **only one** of the following:

- Once
- 2 to 5 times
- 6 to 10 times
- 10 or more times
- I cannot or do not wish to answer this question

89 Since COVID-19 crisis began, has your partner or husband physically forced you to have sexual intercourse or do something sexual when you did not want to?

Please choose **only one** of the following:

- Yes
- No
- I cannot or do not wish to answer this question

90 If yes, did this happen...

Please choose **only one** of the following:

- Before the COVID-19 crisis
- Since the COVID-19 crisis started
- Both before and after the COVID-19 crisis

91 How often did this happen since the COVID-19 crisis began?

Please choose **only one** of the following:

- Once
- 2 to 5 times
- 6 to 10 times
- 10 or more times
- I cannot or do not wish to answer this question

92 Since COVID-19 crisis began, has someone who is **<u>not</u>** your regular partner forced or pressured you into sexual activity?

Please choose **only one** of the following:

- ◯ Yes
- ◯ No
- ◯ I cannot or do not wish to answer this question

93 If yes, did this happen...

Please choose **only one** of the following:

- ◯ Before the COVID-19 crisis
- ◯ Since the COVID-19 crisis started
- ◯ Both before and after the COVID-19 crisis

94 How often did this happen since the COVID-19 crisis began?

Please choose **only one** of the following:

- ◯ Once
- ◯ 2 to 5 times
- ◯ 6 to 10 times
- ◯ 10 or more times
- ◯ I cannot or do not wish to answer this question

95 If yes, who forced or pressured you to have sex in past 6 months?

Please choose **all** that apply:

- ☐ A non-relative who lives with you
- ☐ Uniformed officer (police, military, security officer)
- ☐ Trusted individual (teacher, classmate, friend, co-worker, neighbour, religious leader)
- ☐ Stranger
- ☐ Sex work client
- ☐ Family member (parent, sibling, relative)
- ☐ Other
- ☐ I cannot or do not wish to answer

Please be reassured, all responses are anonymous and confidential

96 Did you report these instances of violence?

Please choose **only one** of the following:

- ◯ Yes
- ◯ No

97 What prevented you from reporting these instances of violence?

Please choose **all** that apply:

- ☐ I could not report the case due to lockdown/ my movements were restricted
- ☐ I don’t know how to report this
- ☐ I was afraid of reporting the case
- ☐ The police will do nothing/ there will be no justice
- ☐ I was embarrassed
- ☐ I could not go to report the case (I was hurt and unable to move)
- ☐ Other

98
Nothing justifies any form of violence to you, never!

Please note or take a screenshot of the numbers and links below:

Hotlines: 08052004698 or 08180091072

Website: http://www.projectalertnig.org (http://www.projectalertnig.org/)
Facebook page: http://www.facebook.com/ProjectAlertOnViolenceAgainstWomen (https://www.facebook.com/ProjectAlertOnViolenceAgainstWomen/)

Citizenship

This section contains 4 questions on citizenship and migrancy status. We assure you that this information is confidential. It is NOT shared with any authority.

99
What is your nationality?

Please choose **only one** of the following:

- ◯ Nigeria
- ◯ Other African country
- ◯ Other non-African country

100
Please be reassured, all your answers are confidential and anonymous. They will NOT be shared with any authority, never.

Would you say you are:

Please choose **only one** of the following:

- ◯ I am a migrant
- ◯ I am a refugee
- ◯ I am seeking asylum
- ◯ I cannot or do not wish to answer
- ◯ I don't know

101 Are you a returning migrant or have you been displaced from your home place?

Please choose **only one** of the following:

- ◯ No, I am not a returning migrant or a displaced person
- ◯ Yes, I am a returning migrant (I migrated to another country and came back here)
- ◯ Yes, I have been forced to move from my home place
- ◯ I don't know
- ◯ I cannot or do not wish to answer this question

102 Why did you leave?

Please choose **all** that apply:

- [ ] Economic reasons (example: job, salary)
- [ ] Family considerations
- [ ] Drought
- [ ] Tribal conflicts
- [ ] Forced relocation
- [ ] Insecurity
- [ ] Violation of human rights
- [ ] Armed conflicts
- [ ] Other: ______

103 Do you have citizenship, or a valid residence permit (e.g. a work permit) for Nigeria?

Please choose **only one** of the following:

- ○ Yes
- ○ No
- ○ Unsure
- ○ I cannot or do not wish to answer

Transactional Sex and Sex Work

This section contains questions about non-marital sexual relationships in exchange for money, material support or other benefit.

Please be reassured again, your answers are confidential and will NOT be told to anyone. You can skip any question you are not comfortable with.

104 In the past 12 months, did you enter into a sexual relationship with a man (NOT your husband) mainly in order to get things that you need, money, gifts, school fees, clothes, or other things that are important to you?

Please choose **only one** of the following:

- ◯ Yes, before the COVID-19 crisis but not now
- ◯ Yes, before the COVID-19 crisis and currently
- ◯ Yes, since the COVID-19 crisis but not before
- ◯ No, I have never
- ◯ I am unsure
- ◯ I do not want to answer

105 The last time you had sexual intercourse with this person from previous question, was a condom used?

Please choose **only one** of the following:

- ◯ Yes
- ◯ No

106
Have you ever engaged in sex work (being paid to have sex)?

Please choose **only one** of the following:

- ◯ Yes, before the COVID-19 crisis but not now
- ◯ Yes, before the COVID-19 crisis and currently
- ◯ Yes, since the COVID-19 crisis but not before
- ◯ No, I have never
- ◯ I am unsure
- ◯ I do not want to answer

107

Has the COVID-19 crisis influenced your engagement in sex work?

Please choose **all** that apply:

- [ ] I engage in more sex work
- [ ] I engage in sex work online
- [ ] I earn less money per customer
- [ ] I take more risks or do things beyond my comfort
- [ ] I engage in more condomless sex
- [ ] I have stopped sex work
- [ ] I engage in less sex work
- [ ] No

108 What is the main reason for engaging in more condomless sex?

Please choose **all** that apply:

- [ ] Condom stockout
- [ ] Afraid to go to the pharmacy
- [ ] Lockdown and curfews make it harder to buy condoms
- [ ] No money to buy condoms
- [ ] Harder to negotiate condom use with clients
- [ ] Other: ____________

109 Has the COVID-19 crisis created additional police crackdowns on sex work where you live?

Please choose **only one** of the following:

- ◯ Yes
- ◯ No
- ◯ Unsure
- ◯ I cannot or do not wish to answer

Alcohol and Substance Use

This section is about alcohol and substance or drug use, as well as the impact of COVID-19 on its consumption.

110 How has your alcohol use changed since the COVID-19 crisis began?

Please choose **only one** of the following:

- ◯ I started drinking during COVID-19 crisis (I was not drinking before)
- ◯ Increased
- ◯ No change
- ◯ Decreased
- ◯ Does not apply to me, I don't drink

This includes ogogoro, palm wine (tombo, pammy, ...), burukutu, pito and other local alcoholic beverages

111

How often have you had a drink containing alcohol since the COVID-19 crisis began, (that is since March 2020)?

Please choose **only one** of the following:

- ◯ Never
- ◯ Monthly or less
- ◯ 2 to 4 times a month
- ◯ 2 to 3 times per week
- ◯ 4 or more times per week

This includes ogogoro, palm wine (tombo, pammy, ...), burukutu, pito and other local alcoholic beverages

112

How many drinks did you have on a typical day when you are drinking?

Please choose **only one** of the following:

- ◯ 1 or 2
- ◯ 3 or 4
- ◯ 5 or 6
- ◯ 7 to 9
- ◯ 10 or more

This includes ogogoro, palm wine (tombo, pammy, ...), burukutu, pito and other local alcoholic beverages

113 How often have you had six or more drinks on one occasion?

Please choose **only one** of the following:

- Never
- Less than monthly
- Monthly
- Weekly
- Daily or almost daily

This includes ogogoro, palm wine (tombo, pammy, ...), burukutu, pito and other local alcoholic beverages

114 Since COVID-19 crisis began, have you used tobacco products?

Please choose **only one** of the following:

- Yes
- No
- I don't know
- I cannot or do not wish to answer

115 How has your tobacco use changed since the COVID-19 crisis began?

Please choose **only one** of the following:

- I started smoking during Covid-19 crisis (I was not smoking before)
- Increased
- No change
- Decreased
- I cannot or do not wish to answer this question

116 Since the COVID-19 crisis began, have you used any substance or drug?

Please choose **only one** of the following:

- ◯ Yes
- ◯ No
- ◯ I don't know
- ◯ I cannot or do not wish to answer

For example: marijuana, opioid, "gutter water", heroine, glue, whoonga, speed, ecstasy, flakka, cocaine, codeine, tramadol, etc...

117 How are you using these substance or drugs?

Please choose **only one** of the following:

- ◯ I inject them (with syringe and needles)
- ◯ I don't inject them, meaning I smoke, snort or swallow them
- ◯ I use both ways, meaning injecting and non-injecting them
- ◯ I cannot or do not wish to answer

118 About the injecting drugs, how often do you inject these drugs?

Please choose **only one** of the following:

- ◯ Everyday
- ◯ Once weekly
- ◯ Two or more times a week
- ◯ Once a month or less

119 How has your use of injectable drugs changed since the COVID-19 crisis began?

Please choose **only one** of the following:

- I started injecting drug during Covid-19 crisis (I was not injecting drug before)
- Increased
- No change
- Decreased
- I cannot or do not wish to answer this question

120 Does the COVID-19 crisis limit your access to the following?

Please choose the appropriate response for each item:

	Yes	No	I don't use
**Access to the drug or substance you inject?**	○	○	○
**Safe injection equipment (needle exchange)**	○	○	○
**Opioid harm reduction (opiate substitution)**	○	○	○

121 Did you share your injecting equipment (example sharing needles)?

Please choose **only one** of the following:

- ◯ Yes
- ◯ No
- ◯ I don't know
- ◯ I cannot or do not wish to answer this question

122 How has COVID-19 crisis changed your sharing of injecting equipment?

Please choose **only one** of the following:

- ◯ I started sharing my injecting equipment during COVID-19 (I was not sharing it before)
- ◯ Increased
- ◯ No change
- ◯ Decreased
- ◯ I cannot or do not wish to answer this question

123 Now about the substances or drugs you swallow, snort or smoke (NOT injecting), how often do you use these drugs?

Please choose **only one** of the following:

- ◯ Everyday
- ◯ Once weekly
- ◯ Two or more times a week
- ◯ Once a month or less
- ◯ I only used it once

124 How has your non-injecting drug use changed since the COVID-19 crisis began?

Please choose **only one** of the following:

- ◯ I started using drug during Covid-19 crisis (I was not using drug before)
- ◯ Increased
- ◯ No change
- ◯ Decreased
- ◯ I cannot or do not wish to answer this question

HIV Stigma

If you live with HIV, you may face stigma. Let’s talk about it

Choose how you feel about each statement. We understand some questions might sound disturbing. Please share your experience. It will help to create a more inclusive environment for everyone.

125 Some people avoid touching me once they know I have HIV

Please choose **only one** of the following:

- ◯ Strongly agree
- ◯ Agree
- ◯ Disagree
- ◯ Strongly disagree
- ◯ I don't know

126 People I care about stopped contacting me after learning I have HIV

Please choose **only one** of the following:

- ◯ Strongly agree
- ◯ Agree
- ◯ Disagree
- ◯ Strongly disagree
- ◯ I don't know

127 I have lost friends by telling them I have HIV

Please choose **only one** of the following:

- ◯ Strongly agree
- ◯ Agree
- ◯ Disagree
- ◯ Strongly disagree
- ◯ I don't know

128 Telling someone I have HIV is risky

Please choose **only one** of the following:

- ◯ Strongly agree
- ◯ Agree
- ◯ Disagree
- ◯ Strongly disagree
- ◯ I don't know

129 I work hard to keep my HIV a secret

Please choose **only one** of the following:

- Strongly agree
- Agree
- Disagree
- Strongly disagree
- I don't know

130
I am very careful whom I tell that I have HIV

Please choose **only one** of the following:

- Strongly agree
- Agree
- Disagree
- Strongly disagree
- I don't know

131 People with HIV are treated like outcasts

Please choose **only one** of the following:

- Strongly agree
- Agree
- Disagree
- Strongly disagree
- I don't know

132 Most people believe a person who has HIV is dirty

Please choose **only one** of the following:

- Strongly agree
- Agree
- Disagree
- Strongly disagree
- I don't know

133 Most people are uncomfortable around someone with HIV

Please choose **only one** of the following:

- Strongly agree
- Agree
- Disagree
- Strongly disagree
- I don't know

134 I feel guilty because I have HIV

Please choose **only one** of the following:

- Strongly agree
- Agree
- Disagree
- Strongly disagree
- I don't know

135 People’s attitudes about HIV make me feel worse about myself

Please choose **only one** of the following:

- Strongly agree
- Agree
- Disagree
- Strongly disagree
- I don't know

136 I feel I’m not as good a person as others because I have HIV

Please choose **only one** of the following:

- Strongly agree
- Agree
- Disagree
- Strongly disagree
- I don't know

137 In the last 6 months, have you ever been excluded from social gatherings or activities (e.g., weddings, funerals, parties, clubs) because of your HIV status?

Please choose **only one** of the following:

- Yes, once
- Yes, several times
- No
- I don't know
- It doesn't apply to me

138 Living with HIV can open new horizons and positively affects us deep inside. Which of the following have been positively affected by your HIV status

Please choose **all** that apply:

- ☐ my self-confidence
- ☐ my self-respect
- ☐ my ability to respect others
- ☐ my ability to cope with stress
- ☐ my ability to better take care of my health
- ☐ my ability to contribute to my community
- ☐
- ☐ Other:

Transgender people

This module is about your needs as a transgender person and how the COVID-19 crisis affected your access to services you need

139 In the past 3 months, has the COVID-19 situation limited your ability to access medications and hormones specific to your trans healthcare?

Please choose **only one** of the following:

- ○ Yes
- ○ No
- ○ I don't use this resource
- ○ I don't know
- ○ I cannot or do not wish to answer this question

140 In the past 3 months, has the COVID-19 situation limited your ability to access to non-medical supplies? (example: make-up, shaving supplies, wigs, breast forms, etc.)

Please choose **only one** of the following:

- Yes
- No
- I don't use this resource
- I don't know
- I cannot or do not wish to answer this question

141 In the past 3 months, has the COVID-19 situation limited your ability to access to therapy or counselling services for transgender-specific support? (including peer support groups)

Please choose **only one** of the following:

- Yes
- No
- I don't use this resource
- I don't know
- I cannot or do not wish to answer this question

142 In the past 3 months, has the COVID-19 situation limited your ability to access gender-affirmation or transition-related surgery?

Please choose **only one** of the following:

- ◯ Yes
- ◯ No
- ◯ I don't use this resource
- ◯ I don't know
- ◯ I cannot or do not wish to answer this question

143
If yes, how was your surgery or surgeries impacted?

Please choose **only one** of the following:

- ◯ Cancelled without rescheduling and/or delayed indefinitely
- ◯ Rescheduled and completed
- ◯ Rescheduled for the future with the same provider
- ◯ Rescheduled for the future with a different provider

End

144
Thank you for completing our survey!

Would you like to give us permission to recontact you on this telephone number in the future for more information or to find out if you would be willing to participate in another survey?

Please choose **only one** of the following:

- ◯ Yes
- ◯ No

145 Please be reassured, your contact will be permanently and irremediably disconnected from your answers

Thank you for your participation!

Submit your survey.
Thank you for completing this survey.
